# Targeted silencing of SOCS1 by DNMT1 promotes stemness of human liver cancer stem-like cells

**DOI:** 10.1186/s12935-024-03322-4

**Published:** 2024-06-12

**Authors:** Lei Lou, Tingyun Deng, Qing Yuan, Lianghou Wang, Zhi Wang, Xiang Li

**Affiliations:** Department of Preclinical Medicine, Hunan Normal University School of Medicine, Changsha, 410013 China

**Keywords:** DNMT1, SOCS1, Hepatocellular carcinoma, Human liver cancer stem-like cells, Stemness

## Abstract

**Background:**

Human liver cancer stem-like cells (HLCSLCs) are widely acknowledged as significant factors in the recurrence and eradication of hepatocellular carcinoma (HCC). The sustenance of HLCSLCs’ stemness is hypothesized to be intricately linked to the epigenetic process of DNA methylation modification of genes associated with anticancer properties. The present study aimed to elucidate the stemness-maintaining mechanism of HLCSLCs and provide a novel idea for the clearance of HLCSLCs.

**Methods:**

The clinical relevance of DNMT1 and SOCS1 in hepatocellular carcinoma (HCC) patients was evaluated through the GEO and TCGA databases. Cellular immunofluorescence assay, methylation-specific PCR, chromatin immunoprecipitation were conducted to explore the expression of DNMT1 and SOCS1 and the regulatory relationship between them in HLCSLCs. Spheroid formation, soft agar colony formation, expression of stemness-associated molecules, and tumorigenicity of xenograft in nude mice were used to evaluate the stemness of HLCSLCs.

**Results:**

The current analysis revealed a significant upregulation of DNMT1 and downregulation of SOCS1 in HCC tumor tissues compared to adjacent normal liver tissues. Furthermore, patients exhibiting an elevated DNMT1 expression or a reduced SOCS1 expression had low survival. This study illustrated the pronounced expression and activity of DNMT1 in HLCSLCs, which effectively targeted the promoter region of SOCS1 and induced hypermethylation, consequently suppressing the expression of SOCS1. Notably, the stemness of HLCSLCs was reduced upon treatment with DNMT1 inhibitors in a concentration-dependent manner. Additionally, the overexpression of SOCS1 in HLCSLCs significantly mitigated their stemness. The knockdown of SOCS1 expression reversed the effect of DNMT1 inhibitor on the stemness of HLCSLCs. DNMT1 directly binds to the SOCS1 promoter. In vivo, DNMT1 inhibitors suppressed SOCS1 expression and inhibited the growth of xenograft.

**Conclusion:**

DNMT1 targets the promoter region of SOCS1, induces hypermethylation of its CpG islands, and silences its expression, thereby promoting the stemness of HLCSLCs.

**Supplementary Information:**

The online version contains supplementary material available at 10.1186/s12935-024-03322-4.

## Introduction

Hepatocellular carcinoma (HCC) is one of the ten cancers with the highest number of new cases and deaths worldwide each year, accounting for 8.3% of all cancer deaths [1]. Thus, eradicating HCC would be effective in alleviating the global cancer burden. Human liver cancer stem-like cells (HLCSLCs) are characterized by stemness, tumor initiation, strong self-renewal, and high heterogeneity [2–4]. The presence of such cells is the biological basis for the rapid progression, recurrence, multi-drug resistance, and high mortality of HCC [5, 6]. Hence, the mechanism of stemness maintenance of HLCSLCs has been under intensive focus. Several studies have shown that the stemness of HLCSLCs can be evaluated from various aspects, such as spheroid formation, soft agar colony formation, expression of stemness-associated molecules, and tumorigenicity of xenograft in nude mice [7–9]. CD44, Oct4, Nanog, and Sox2 have been frequently used as stemness-associated molecules in HLCSLCs [10–12]. Lin et al. speculated that Zinc-finger and homeobox 2 (ZHX2) inhibits HCC by suppressing stem cell-like traits through lysine demethylase 2 A (KDM2A)-mediated histone H3 lysine 36 (H3K36) demethylation [13]. Wang et al. showed that the hypermethylation of tumor-suppressive miR-148a promotes the stemness of HLCSLCs, and arsenic trioxide (ATO) activates miR-148a by inducing DNA demethylation, thus inhibiting the CSC-like phenotype [14]. These findings suggested that epigenetic modifications, especially hypermethylation of tumor suppressor genes, maintain the stemness in HLCSLCs.

DNA methyltransferase 1 (DNMT1) is a critical enzyme for maintaining the DNA methylation status in mammals and is highly expressed in various tumor tissues, including HCC [15–18]. Importantly, the upregulated expression of DNMT1 can partially rescue the stemness of CD133^+^/CD44^+^ subgroup in HCC, thus helping these cells to resist apoptosis or differentiation [19]. Wang et al. reported that brain-expressed X-linked protein 1 (BEX1) could be used as a new marker in the self-renewal of hepatocellular carcinoma stem cells (CSCs). DNMT1 inhibitors induce BEX1 expression by eliminating the epigenetic repression of BEX1 by DNMT1 [20]. This phenomenon suggested a critical role of the epigenetic reprogramming mechanism of DNA methylation in stem cell biology. In HCC cells, DNMT1 can target binding to the anticancer gene *p53*, induce hypermethylation of its promoter, partially silence p53 expression, and promote HCC cell proliferation and invasion ability [21], suggesting the role of DNMT1 in the hypermethylation of promoter CpG island of the tumor suppressor gene. Subsequently, the tumor suppressor gene is silenced and cancer progression is induced.

Suppressor of the cytokine signaling 1 (SOCS1) is one of the eight members of the SOCS family of transcription factors. As an inhibitory molecule of the JAK-STAT signaling pathway, SOCS1 has attracted significant attention. It has a low expression in a variety of tumors, including HCC, head and neck cancers, and osteosarcoma, and is considered a novel tumor suppressor gene [22–24]. Sun et al. proposed that oxidative stress decreases the expression of SOCS1 in HCC and the activation of STAT3, increasing cell migration and invasion, while antioxidant treatment reversed this process [25]. Liu et al. concluded that HCV infection has a direct inhibitory effect on SOCS1 expression and that SOCS1 might be involved in HCV infection-induced HCC [26]. In addition, the hypermethylation of CpG islands and low expression of SOCS1 was observed in both HCC tumor samples and HCC cell lines [27]. The removal of SOCS1 hypermethylation and SOCS1 restoration was effective in HLCSLCs when different subtypes of HCC cells were treated with DNMT1 inhibitors [28]. These phenomena suggested that the low expression of SOCS1 in HCC may be related to epigenetic modifications.

In summary, a correlation was established between the epigenetic enzyme DNMT1 and the promoter hypermethylation of the tumor suppressor gene *SOCS1* in the progression of HCC; however, evidence on whether this epigenetic regulation is involved in the maintenance of stemness in HLCSLCs is yet lacking. The current study demonstrated that DNMT1 targeted the downregulation of SOCS1 using DNA methylation modification in vitro and in vivo to maintain the high stemness of HLCSLCs, including high spheroid formation and soft agar colony-forming capacity, elevated expression of stemness-associated molecules, and strong tumorigenicity. Together, these findings suggested a novel mechanism for stemness maintenance in HLCSLCs, which provided new targets and strategies for eliminating HLCSLCs in HCC.

## Materials and methods

### Cells and reagents

Human HCC cell lines MHCC97H and SK-Hep-1 were acquired from Beina Chuangli Biotechnology (Beijing, China) and cultured in DMEM medium containing 10% fetal bovine serum (FBS) in an incubator at 37 °C and 5% CO_2_. The second-generation spheres of MHCC97H or SK-Hep-1 cells were obtained using sphere-forming assay and were considered as HLCSLCs used in follow-up experiments. The results of HLCSLCs identification are shown in Figure S1.

### Cellular immunofluorescence assay

An equivalent of 5 × 10^4^ cells/well were cultured in a 12-well plate on coverslips, fixed with 4% paraformaldehyde for 15 min, permeabilized with phosphate-buffered saline (PBS) solution containing 0.2% TritonX-100 for 10 min, and incubated with 1% freshly configured goat serum at room temperature for 1 h to prevent nonspecific binding. Then, the cells were incubated with the primary antibody at 4°C overnight and then with the corresponding fluorescent secondary antibody at room temperature for 1 h. The nuclei were stained with anti-fluorescence attenuating tablets containing 4, 6-diamidino-2-phenylindole (DAPI). The images were acquired with Leica TCS SP8 SR confocal microscopy (Olympus, Germany).

### Methylation-specific PCR (MSP)

Genomic DNA was extracted from HCC cells using the TIANamp Genomic DNA Kit (DP304, Tiangen), following the manufacturer’s protocol. DNA Bisulfite Conversion Kit (DP215, Tiangen) was used to configure the bisulfite reaction system according to the manufacturer’s instructions, and PCR was used for conversion. Methylation-specific PCR Kit (EM101, Tiangen) was used to amplify 400 bp fragments using bisulfite-treated genomic DNA as a template in a 20 µl volume. The bisulfite reaction system is shown in Tables S1. The primer design, MSP reaction system and thermal cycling conditions are displayed in Tables S2-4. The PCR product (10 µl) was analyzed by agarose gel electrophoresis.

### Bisulfite sequencing PCR (BSP)

The suspension containing 1 × 10^6^ cells was centrifuged at 1,000 rpm for 5 min, and the cellular genomic DNA was extracted. 20 µL of genomic DNA sample was taken in a PCR tube, and a bisulfite reaction system was prepared for PCR amplification. 1×TAE, 2.0% agarose, 4 V/cm electrophoresis was performed, and purify DNA from agarose gel. The purified DNA is used in the ligation reaction. The ligation product was added to the competent cells, LB medium was added, and the culture medium was aspirated and spread on LB plates and incubated overnight at 37℃. Take the positive colonies on the plate, inoculate them into liquid medium at 37℃ with shaking, electrophoresis the PCR products of the bacterial liquid, and select the bacterial liquid with correct size of electrophoresis bands for sequencing.

### Western blot analysis

RIPA lysis buffer and PMSF were used to extract proteins from the cells. An equivalent of total protein was separated by 10% sodium dodecyl sulfate-polyacrylamide gel electrophoresis (SDS-PAGE) and transferred to the PVDF membrane. The membrane was blocked with 5% skim milk in TBST for 2 h and probed with primary antibodies at 4 °C overnight. Subsequently, the membrane was incubated with the corresponding secondary antibody at room temperature for 1 h. The immunoreactive bands were developed using ECL substrate (BMU102-CN, Abbkine). All the antibodies were from Cell Signaling Technology: Oct4 antibody (1:2500, 2788 S), CD44 antibody (1:2500, 3570 S), Nanog antibody (1:2500, 3580 S), Sox2 antibody (1:2500, 4195 S), and beta-actin antibody (1:2500, 8157 S).

### Chromatin immunoprecipitation (ChIP)

The cells were detected using the ChIP Assay Kit (P2078, Beyotime). Briefly, the cells were fixed with formaldehyde, counted, dispersed into 1 × 10^6^ cells/tube, washed with PBS containing PMSF, and lysed in SDS lysis buffer on ice for 10 min. Then, the lysate was sonicated for 10 cycles of 1 s power-on and 10 s intervals with the intensity of 30 W. After dilution buffer, Protein A + G was added to the solution. Rabbit IgG and DNMT1 antibody (2 µg) were added sequentially to the mixture and incubated at 4 ℃ overnight. On the second day, the elution buffer was incubated at 65 ℃ for 4 h. After DNA purification, the samples were amplified by PCR, and the amplicons were detected by agarose gel electrophoresis. The reaction system and primer design are shown in Tables S5 and S6. The antibodies were as follows: DNMT1 antibody (1:100, 5032 S, Cell Signaling Technology) and SOCS1 antibody (1:100, ABP52477, Abbkine).

### Lentivirus infection

The interference sequence was designed according to the *SOCS1* gene nucleotide sequence provided by the GenBank database (NM_003745.2). The SOCS1-siRNA was designed and synthesized from Tsingke Biotechnology Co., Ltd (Table S7), and the recombinant lentivirus packaged with SOCS1 overexpression vector was provided by GenePharma. An equivalent of 5 × 10^4^ HCC cells/well were inoculated into 6-well plates and transfected using Lipofectamine 2000, according to the manufacturer’s instructions, when the cell confluency was 70–80%, and the medium was changed 4–6 h post-transfection. The resistant cells are obtained after puromycin (4 µg/mL) screening, and these cells are collected as established new cell lines.

### Spheroid formation assay

An equivalent of 5000 HCC cells/well was inoculated into the Ultra-Low attachment 6-well plates and cultured in stem cell medium at 37 °C under 5% CO_2_. The media were refreshed every 2–3 days, and the tumor lesions were counted and images captured after 2 weeks.

### Soft-agar colony formation assay

1.2% and 0.7% agarose solution was prepared in a constant temperature water bath at 50 °C. Layer agar: 1.2% soft agar was mixed with DMEM containing 20% FBS at 1:1. A volume of 2 mL was evenly spread on the wells and allowed to solidify at room temperature. Cell suspension (1 × 10^3^ HCC cells/mL) was mixed with 0.7% agarose in equal proportion and quickly added to the wells. After 2 weeks, the plates were stained with 0.05% crystal violet. The colonies were counted and images captured. The colony formation rate = number of colonies/number of inoculated cells.

### Tumor xenograft model

All animal experiments were approved by the Ethics Committee of Hunan Normal University and the Committee of Experimental Animal Feeding and Management (Permit No: 2020 − 272). 3-4-week-old BALB/c female and male nude mice were purchased from Hunan SJA Laboratory Animal Co., Ltd and quarantined (domesticated) for a week before the experiments. A mixture containing 5 × 10^5^ HLCSLCs, culture medium, and Matrigel was injected subcutaneously in nude mice (two injection sites per mouse). The treatment experiment of xenografts was started when the size of the tumors approached 100 mm^3^. The animals were divided into four groups: control, DAC low concentration (1 mg/kg), DAC medium concentration (5 mg/kg), and DAC high concentration (10 mg/kg). Three mice in each group were injected intraperitoneally every 3 days. After 30 days, nude mice were anesthetized by intraperitoneal injection of 200 µL of 1% sodium pentobarbital and euthanized. The xenograft tumors were excised, the volume and weight recorded and then fixed with 4% paraformaldehyde for immunohistochemical analysis.

### Statistical analysis

The data were analyzed using GraphPad Prism 8.0 software. The experiments were repeated at least three times independently, and the results were displayed as mean ± standard deviation (SD). One-way analysis of variance (ANOVA) or Student’s t-tests were applied to evaluate the differences among the groups. *p* < 0.05 was considered statistically significant.

## Results

### DNMT1 and SOCS1 expression in HCC tissues and the impact on prognosis

**Fig. 1:**
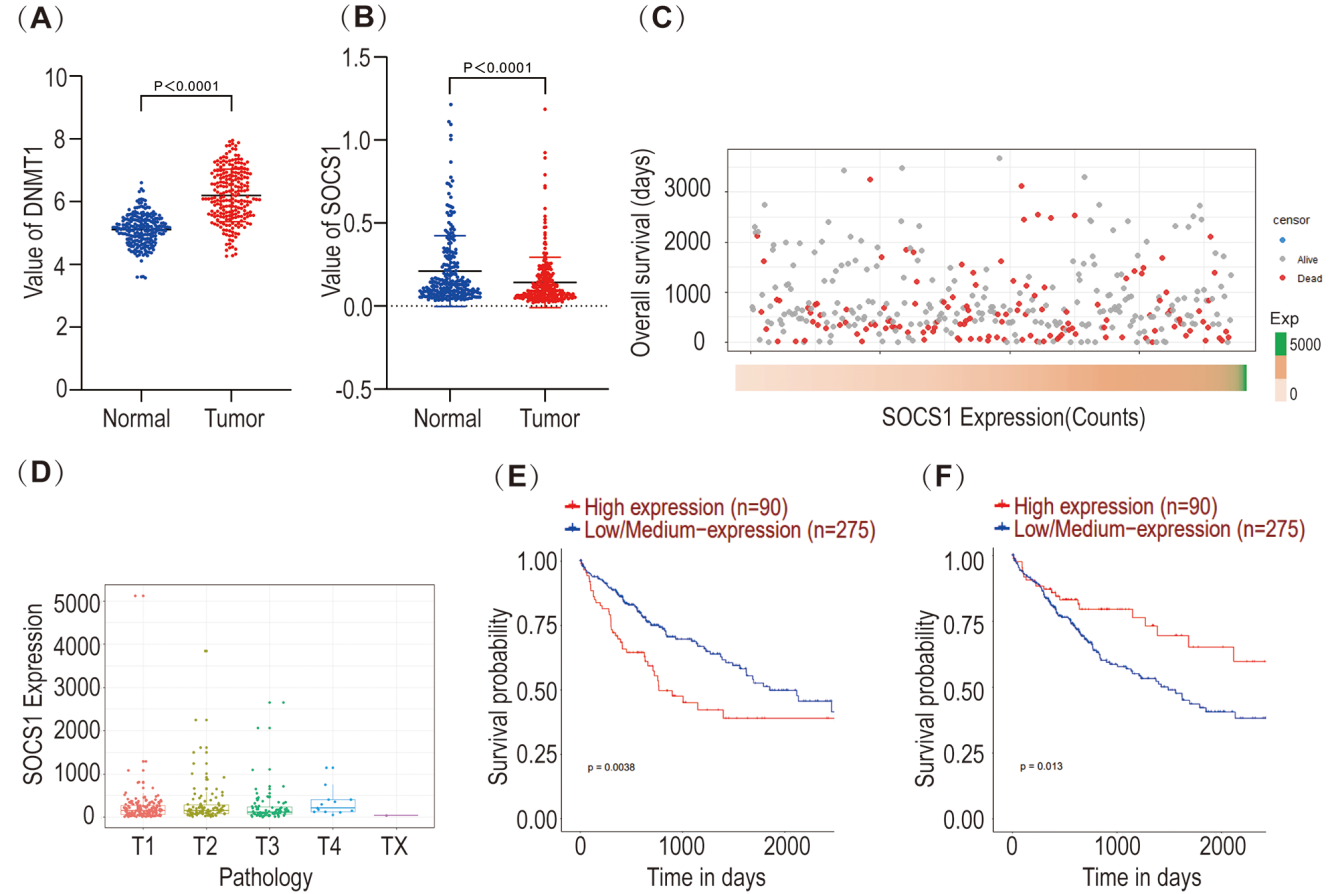
DNMT1 and SOCS1 expression in HCC tissues and the impact on prognosis. (**A**) HCC microarray (GSE14520) in the GEO database was used to compare the difference in expression of DNMT1 in HCC tumor tissues and adjacent normal liver tissues. (**B**) HCC microarray (GSE25097) in the GEO database was used to compare the expression difference of SOCS1 in HCC tumor tissues and adjacent normal liver tissues. (**C**) Overall survival and SOCS1 expression scatterplot (TCGA) of HCC patients. (**D**) Analysis of TNM stage and SOCS1 expression difference in HCC patients. (**E**) Kaplan–Meier curves indicate the overall survival curves of HCC patients (TCGA) with high DNMT1 and low DNMT1 expression. (**F**) Kaplan–Meier curves indicate the overall survival curves of HCC patients (TCGA) with high and low SOCS1 expression

HCC microarrays (GSE14520 and GSE25097) from the GEO database were used to compare the differences in the expression of DNMT1 and SOCS1 in HCC tumor tissues and adjacent normal liver tissues; the results showed that DNMT1 was highly expressed and SOCS1 was lowly expressed in HCC tumor tissues (Fig. 1A, B). Subsequently, we downloaded the genetic and clinical data of 371 HCC patients from the TCGA database (TCGA-LIHC), organized and analyzed the data by RStudio, and plotted the data using ggplot2 (3.4.1), which suggested that the overall survival and TNM stage of 371 HCC patients were asymmetrically distributed with SOCS1 expression (Fig. 1C, D). Therefore, additional intuitive data are essential to elucidate the correlation between SOCS1 and DNMT1 and the prognosis of HCC patients. Next, we analyzed the survival curves of SOCS1 and DNMT1 through UALCAN (based on TCGA-LIHC) and found that patients with high expression of DNMT1 or low expression of SOCS1 had low survival rates in a time-dependent manner (Fig. 1E, F).

### Expression and activity of DNMT1 in HLCSLCs

**Fig. 2:**
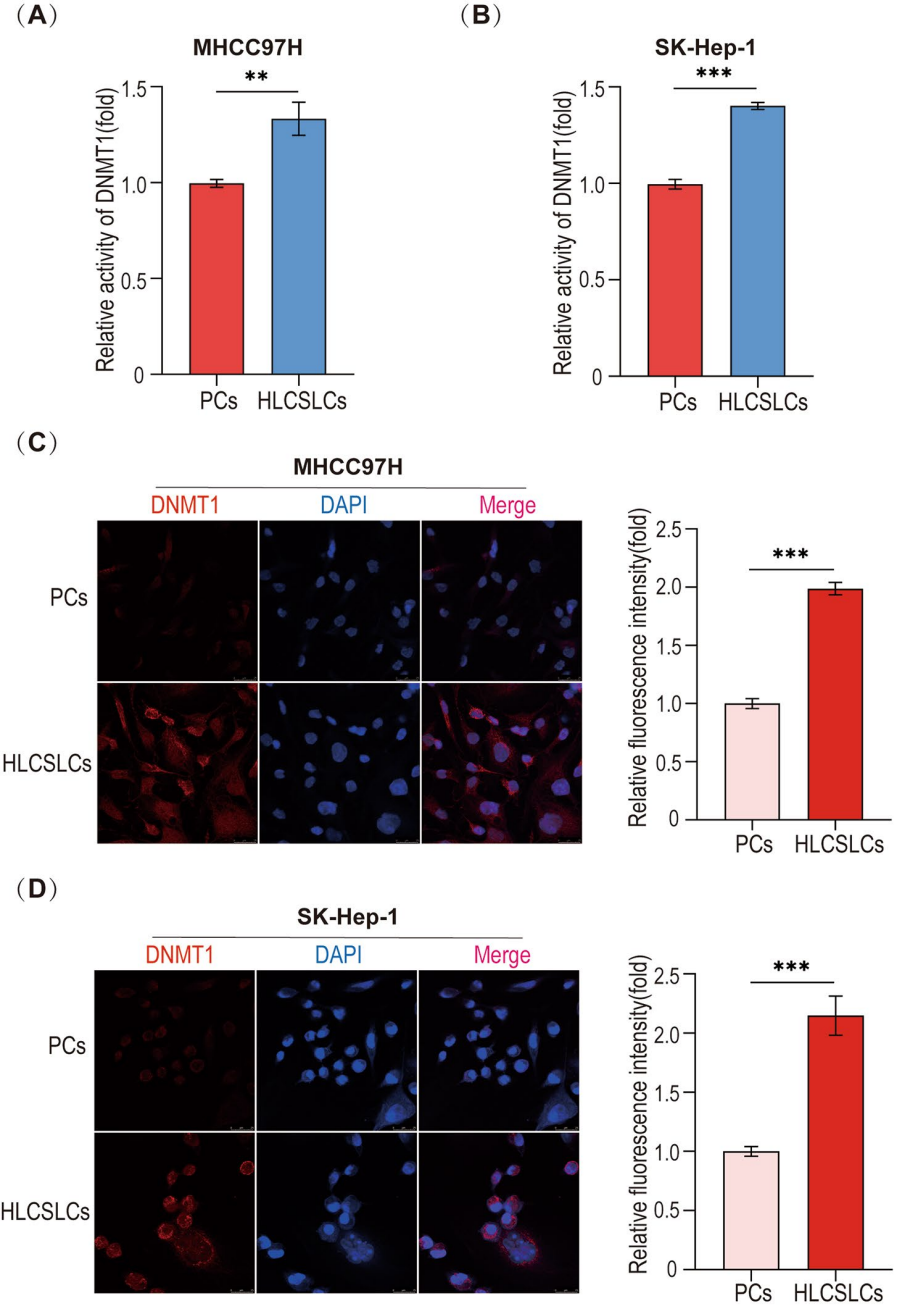
Expression and activity of DNMT1 in HLCSLCs. (**A**) The HCC parental cells (MHCC97H) and HLCSLCs of the corresponding parental origin were assayed for DNMT1 activity. (**B**) The HCC parental cells (SK-Hep-1) and HLCSLCs of the corresponding parental origin were assessed for DNMT1 activity. (**C**) DNMT1 expression levels in MHCC97H parental cells and MHCC97H-derived HLCSLCs were determined by cellular immunofluorescence analysis. (**D**) DNMT1 expression levels in SK-Hep-1 parental cells and SK-Hep-1-derived HLCSLCs were determined by cellular immunofluorescence analysis. **p ≤ 0.01, ***p ≤ 0.001

We observed the expression of DNMT1 in HLCSLCs using cellular immunofluorescence assay and DNMT1 activity assay. The results showed that compared to HCC parental cells (MHCC97H and SK-Hep-1), the expression of DNMT1 in HLCSLCs of the corresponding parental origin was significantly higher and more active (Fig. 2A–D), suggesting that the high expression of DNMT1 may be related to the biological properties of HLCSLCs.

### SOCS1 promoter status and its expression in HLCSLCs

**Fig. 3:**
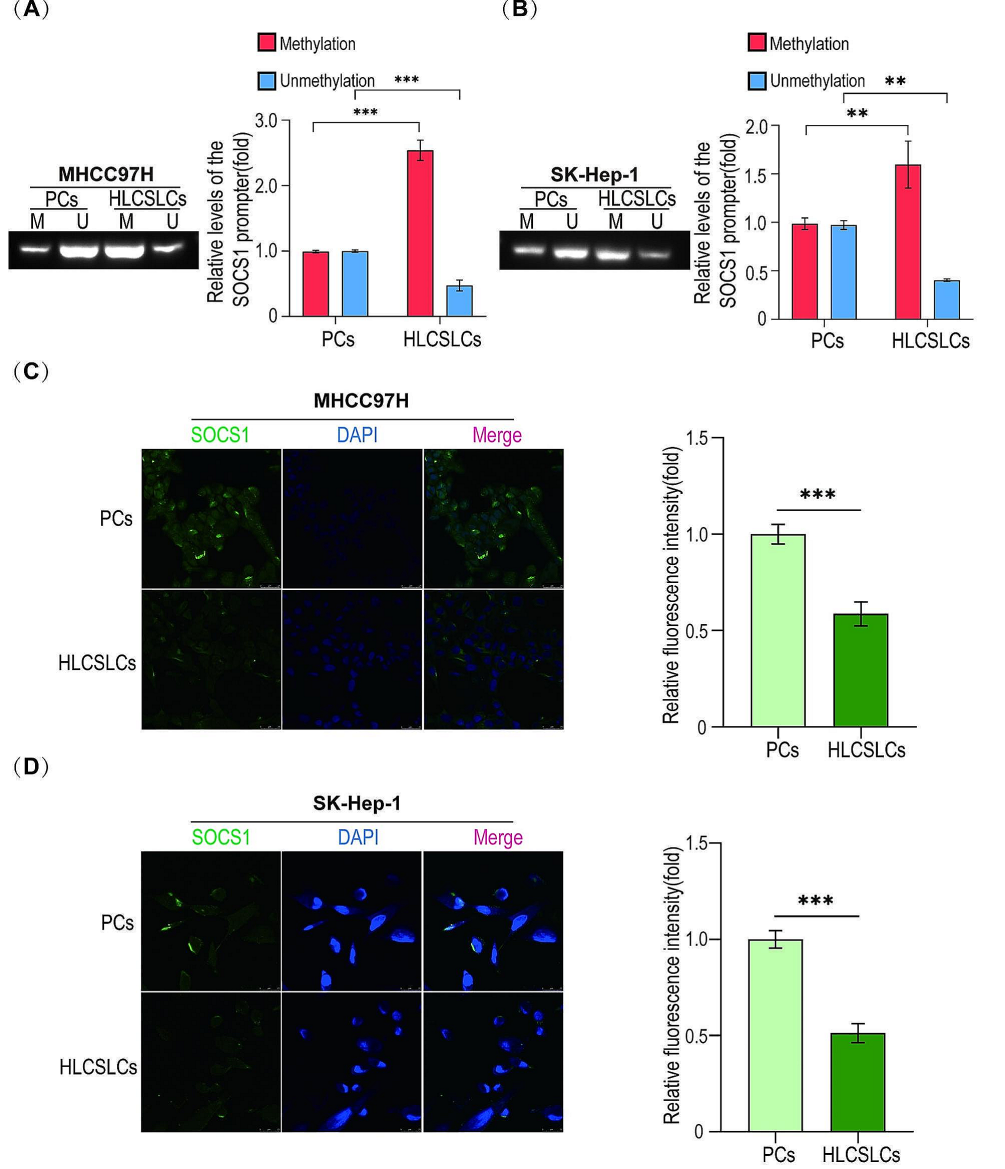
SOCS1 promoter status and its expression in HLCSLCs. (**A**) The methylation status of SOCS1 promoter in MHCC97H parental cells and MHCC97H-derived HLCSLCs was determined by MSP. (**B**) The methylation status of SOCS1 promoter in SK-Hep-1 parental cells and SK-Hep-1-derived HLCSLCs was determined by MSP. (**C**) SOCS1 expression levels in MHCC97H parental cells and MHCC97H-derived HLCSLCs were determined by cellular immunofluorescence analysis. (**D**) SOCS1 expression levels in SK-Hep-1 parental cells and SK-Hep-1-derived HLCSLCs were determined by cellular immunofluorescence analysis. **p ≤ 0.01, ***p ≤ 0.001

We first examined the status of SOCS1 promoter and the expression of the molecule in HLCSLCs by MSP and cellular immunofluorescence assay. The results showed that compared to the parental cells of HCC (MHCC97H and SK-Hep-1), the SOCS1 promoter showed high methylation (Fig. 3A, B) and low SOCS1 expression (Fig. 3C, D) in HLCSLCs. Next, we carried out BSP experiments and the results were consistent with MSP experiments (Figure S2A, B). This finding suggested that low expression of SOCS1 may be associated with the biological properties of HLCSLCs.

### DNMT1 targets the SOCS1 promoter region to regulate SOCS1 expression

**Fig. 4:**
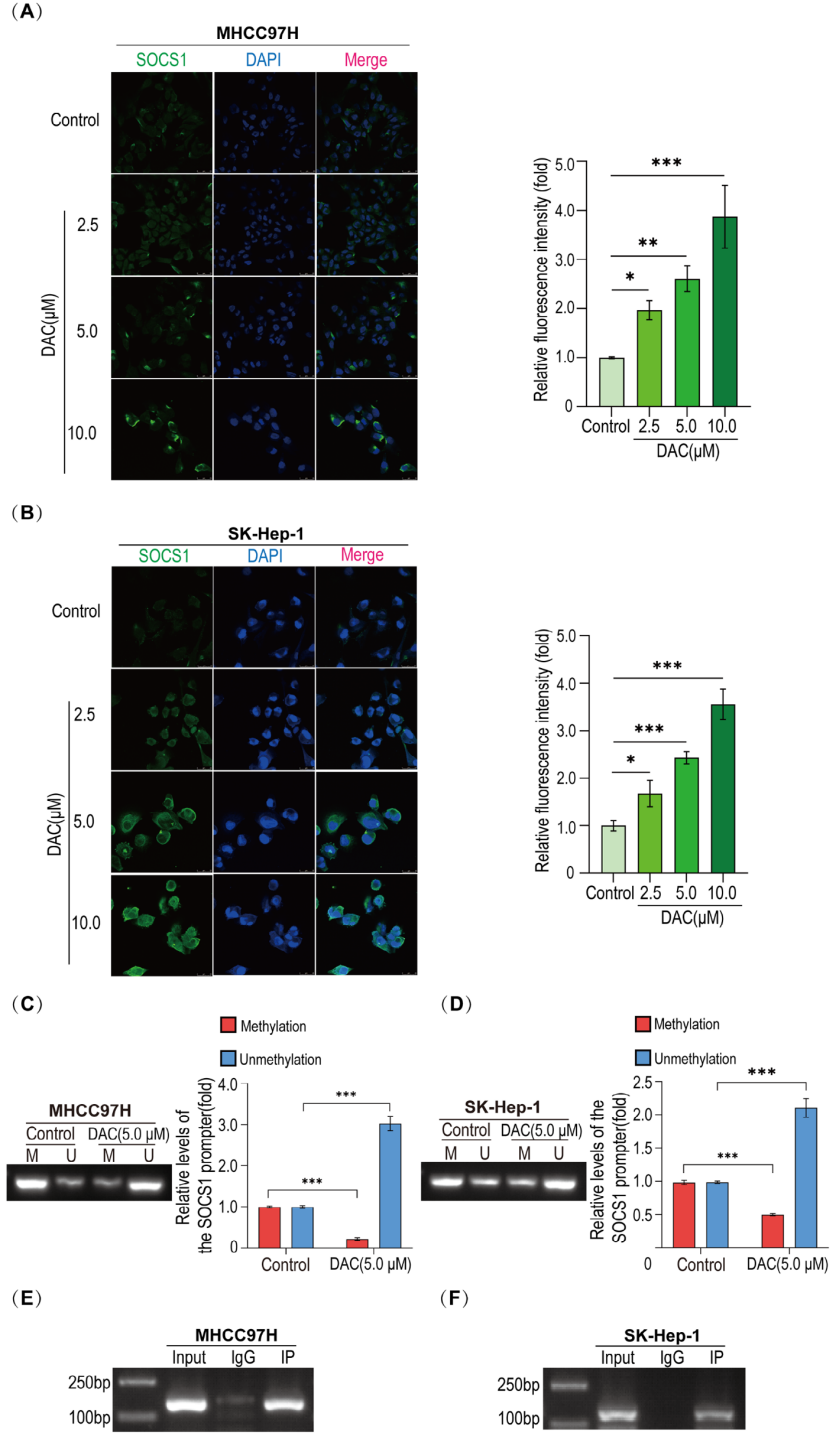
DNMT1 targets the SOCS1 promoter region to regulate SOCS1 expression. (**A**) MHCC97H-derived HLCSLCs treated with different concentrations of DNMT1 inhibitor (DAC) were assayed for SOCS1 expression levels by cellular immunofluorescence analysis. (**B**) SK-Hep-1-derived HLCSLCs treated with different concentrations of DAC were assayed for SOCS1 expression levels by cellular immunofluorescence analysis. (**C**) The methylation status of SOCS1 promoter in MHCC97H-derived HLCSLCs treated with DAC (5.0 µM) was determined by MSP. (**D**) The methylation status of SOCS1 promoter in SK-Hep-1-derived HLCSLCs treated with DAC (5.0 µM) was determined by MSP. (**E**) ChIP assay was used to clarify the binding of DNMT1 and SOCS1 in MHCC97H-derived HLCSLCs. (**F**) ChIP assay was used to clarify the binding of DNMT1 and SOCS1 in SK-Hep-1-derived HLCSLCs. *p ≤ 0.05, **p ≤ 0.01, ***p ≤ 0.001

In order to clarify whether the low expression of SOCS1 is related to the high expression of DNMT1, we treated HLCSLCs with different concentrations of DNMT1 inhibitor. The results of the cellular immunofluorescence assay showed that DNMT1 inhibitor reverses the low expression of SOCS1 in a concentration-dependent manner (Fig. 4A, B), and the results of the MSP assay showed that the hypermethylation of SOCS1 promoter was reduced in the DNMT1 inhibitor (5.0 µM) treated group compared to the control group (Fig. 4C, D). The results of the BSP assay were consistent with those of the MSP detection (Figure S3). The results of the ChIP assay showed that DNMT1 binds to the SOCS1 promoter directly (Fig. 4E, F) and catalyzes its hypermethylation in HLCSLCs, thereby inhibiting the expression of SOCS1. This phenomenon is associated with the maintenance of the biological properties of HLCSLCs.

### Effect of DNMT1 inhibitor on the stemness of HLCSLCs

**Fig. 5:**
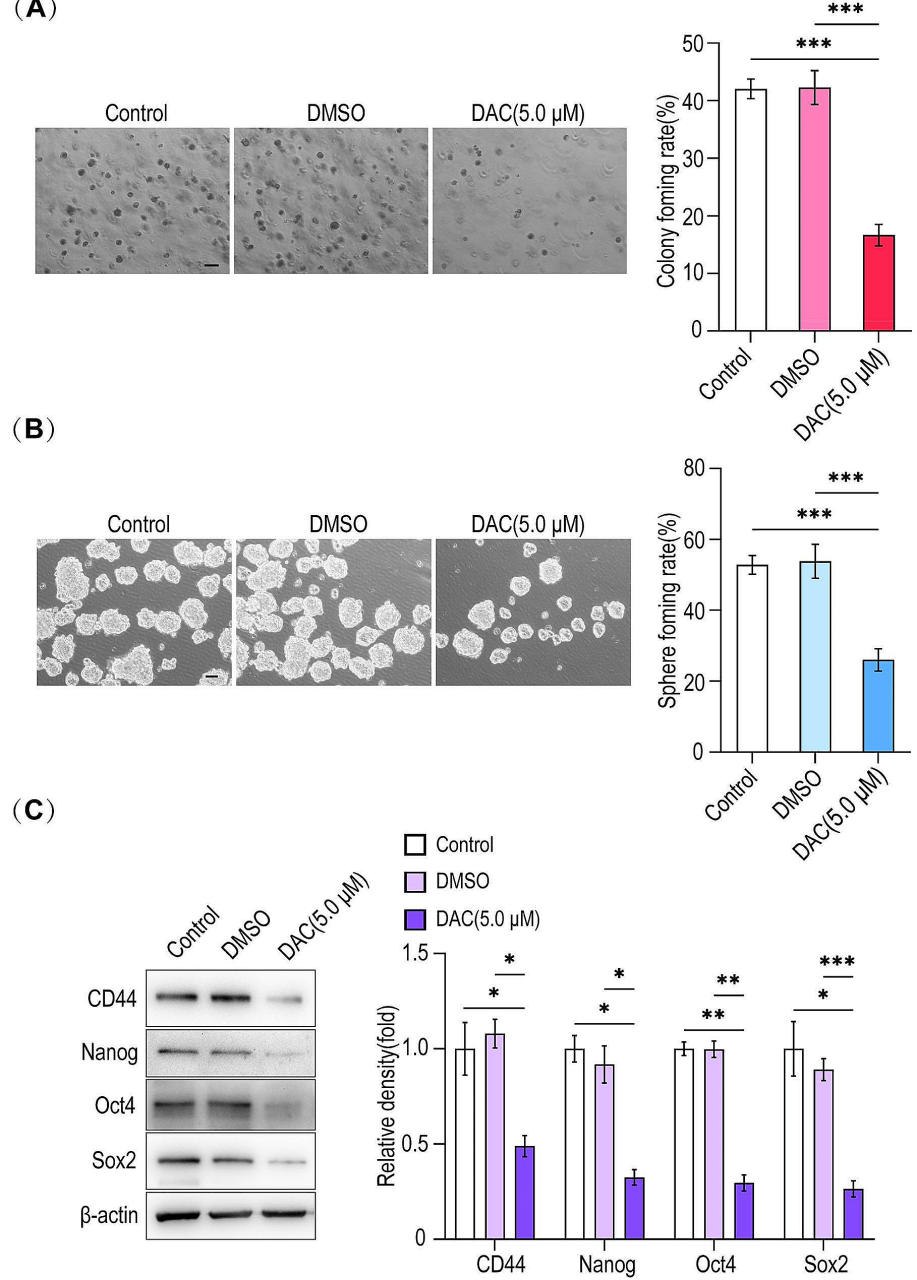
Effect of DNMT1 inhibitor on stemness of HLCSLCs. (**A**) MHCC97H-derived HLCSLCs treated with DAC (5.0 µM) were identified with respect to colony formation. (**B**) MHCC97H-derived HLCSLCs treated with DAC (5.0 µM) had spheroid formation ability. (**C**) The expression of stemness-associated factors (CD44, Oct4, Nanog, and Sox2) was determined by Western blot analysis in MHCC97H-derived HLCSLCs treated with DAC (5.0 µM). *p ≤ 0.05, **p ≤ 0.01, ***p ≤ 0.001

In order to confirm the effect of DNMT1 on the maintenance of stemness of HLCSLCs, we treated the cells with DNMT1 inhibitor (5.0 µM) and observed the changes in their stemness. The results revealed a significantly weakened ability of spheroid and soft agar colony formation of treated HLCSLCs (Fig. 5A, B), and the expression of stemness-associated factors (CD44, Oct4, Nanog, and Sox2) was reduced compared to that of the untreated cells (Fig. 5C). These results suggested that DNMT1 is involved in the maintenance of stemness of HLCSLCs.

### Effect of overexpression of SOCS1 on the stemness of HLCSLCs

**Fig. 6:**
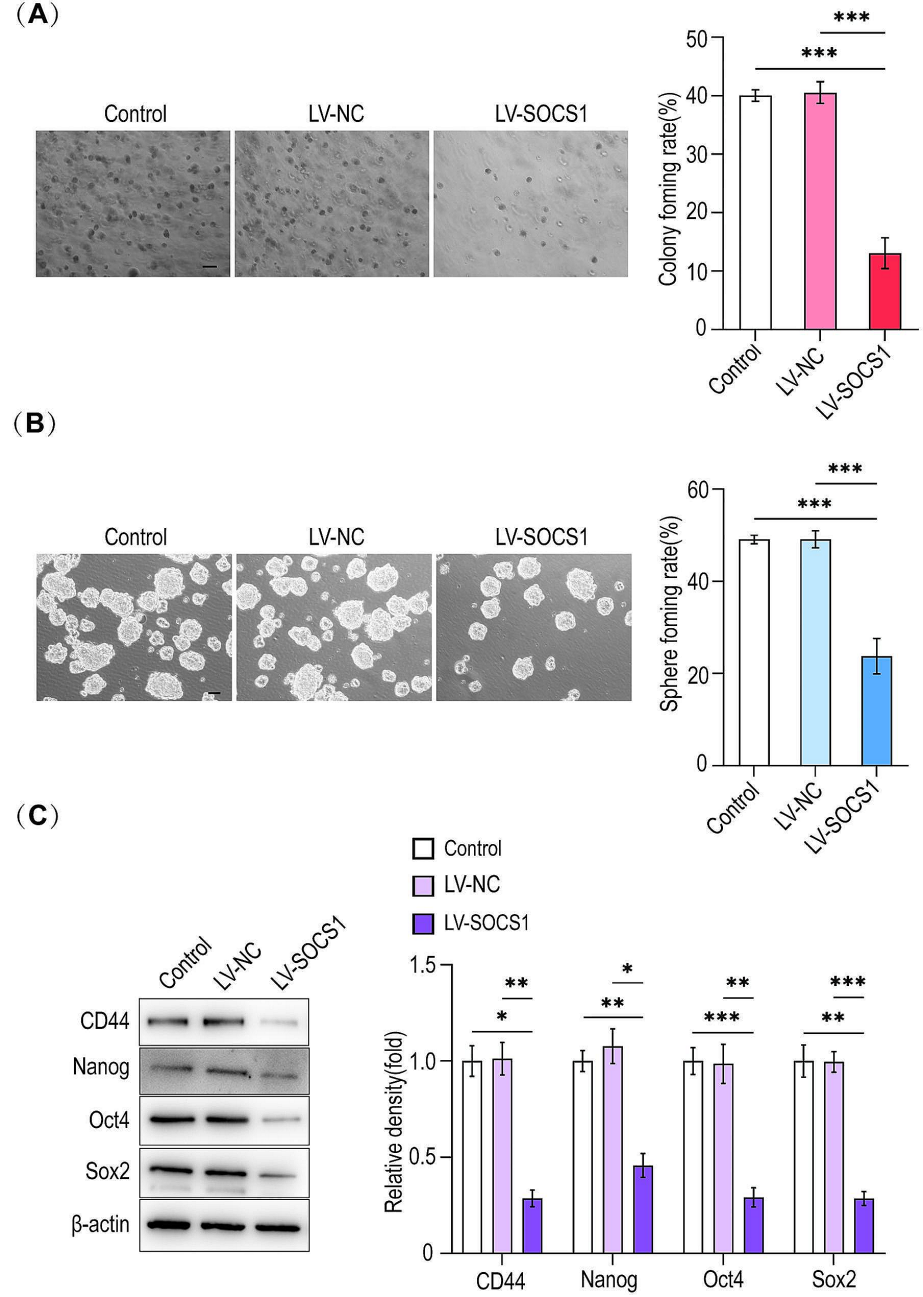
Effect of overexpression of SOCS1 on stemness of HLCSLCs. (**A**) MHCC97H-derived HLCSLCs treated with overexpressed SOCS1 were identified for the ability of colony formation. (**B**) MHCC97H-derived HLCSLCs treated with overexpressed SOCS1 were identified with spheroid formation ability. (**C**) The expression of stemness-associated factors (CD44, Oct4, Nanog, and Sox2) were determined by Western blot analysis in MHCC97H-derived HLCSLCs treated with overexpressed SOCS1. *p ≤ 0.05, **p ≤ 0.01, ***p ≤ 0.001

In order to confirm the effect of SOCS1 on the maintenance of the stemness of HLCSLCs, we overexpressed SOCS1 by lentiviral transfection and observed the changes in the stemness of HLCSLCs. The results showed that compared to the control group, the overexpression of SOCS1 markedly reduced the sphere- and soft agar colony-forming abilities of HLCSLCs (Fig. 6A, B) and the expression of stemness-associated factors (CD44, Oct4, Nanog, and Sox2) (Fig. 6C). These findings suggested that SOCS1 is involved in the maintenance of stemness of HLCSLCs.

### Effect of DNMT1 inhibitor combined with SOCS1-siRNA on the stemness of HLCSLCs

**Fig. 7:**
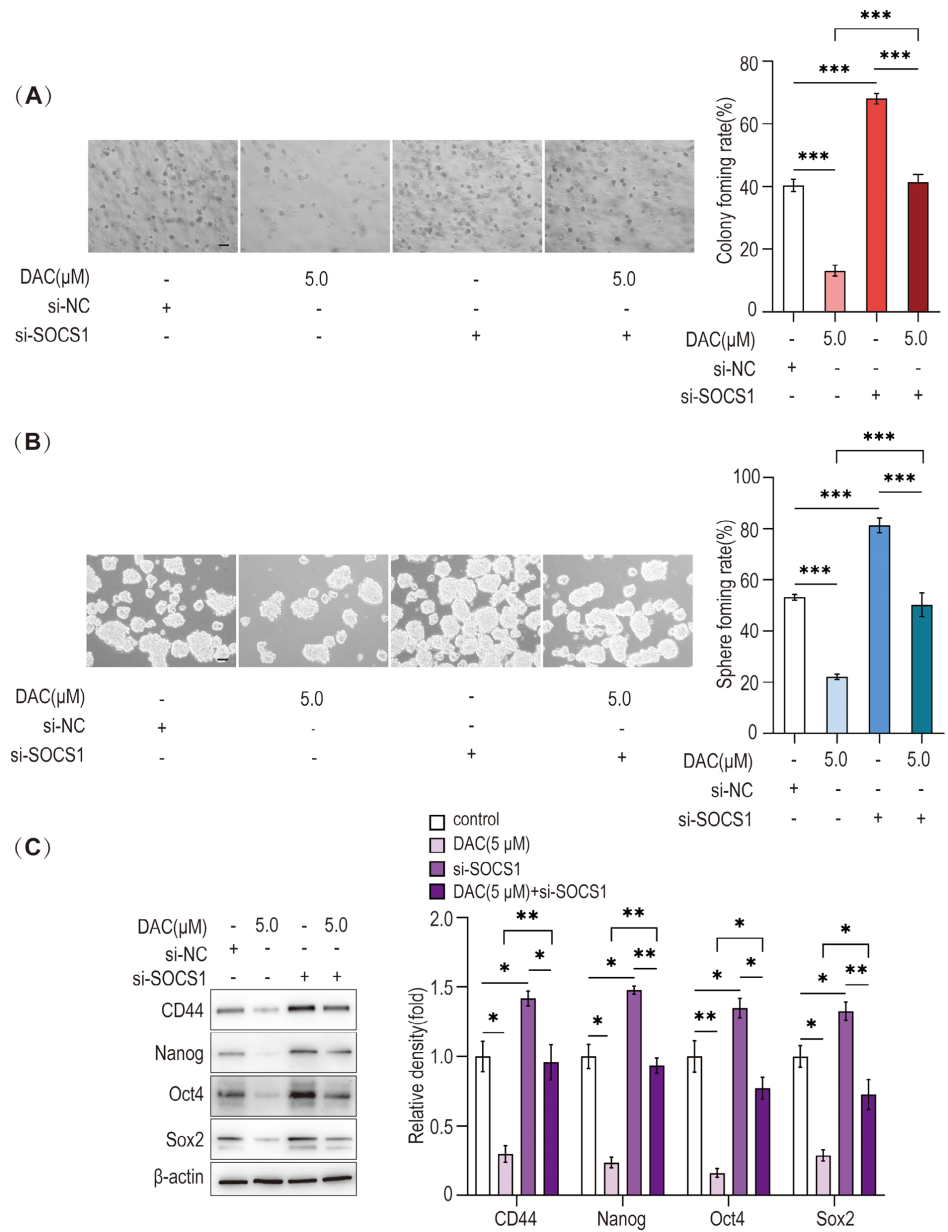
Effect of DNMT1 inhibitor combined with SOCS1-siRNA on the stemness of HLCSLCs. (**A**) MHCC97H-derived HLCSLCs treated with DAC (5.0 µM) combined with SOCS1-siRNA were identified for the ability of colony formation. (**B**) MHCC97H-derived HLCSLCs treated with DAC (5.0 µM) combined with SOCS1-siRNA were identified for spheroid formation ability. (**C**) The expression of stemness-associated factors (CD44, Oct4, Nanog, and Sox2) as determined by Western blot analysis in MHCC97H-derived HLCSLCs treated with DAC (5.0 µM) combined with SOCS1-siRNA. *p ≤ 0.05, **p ≤ 0.01, ***p ≤ 0.001

In order to investigate whether DNMT1 maintains the stemness of HLCSLCs by regulating SOCS1, we first transfected SOCS1-siRNA into HLCSLCs and then treated the cells with DNMT1 inhibitor (5.0 µM). The results showed that compared to the control group, transfection with SOCS1-siRNA alone significantly enhances the ability of HLCSLCs to form spheres and soft agar colonies and increases the expression of stemness-associated factors (CD44, Oct4, Nanog, and Sox2). On the other hand, the role of SOCS1-siRNA in enhancing the stemness of HLCSLCs was attenuated by DNMT1 inhibitor treatment combined with SOCS1-siRNA (Fig. 7A–C). These results suggested that DNMT1 maintains the stemness of HLCSLCs by downregulating SOCS1 expression.

### Effect of DNMT1 inhibitors on the growth of xenograft in nude mice

**Fig. 8:**
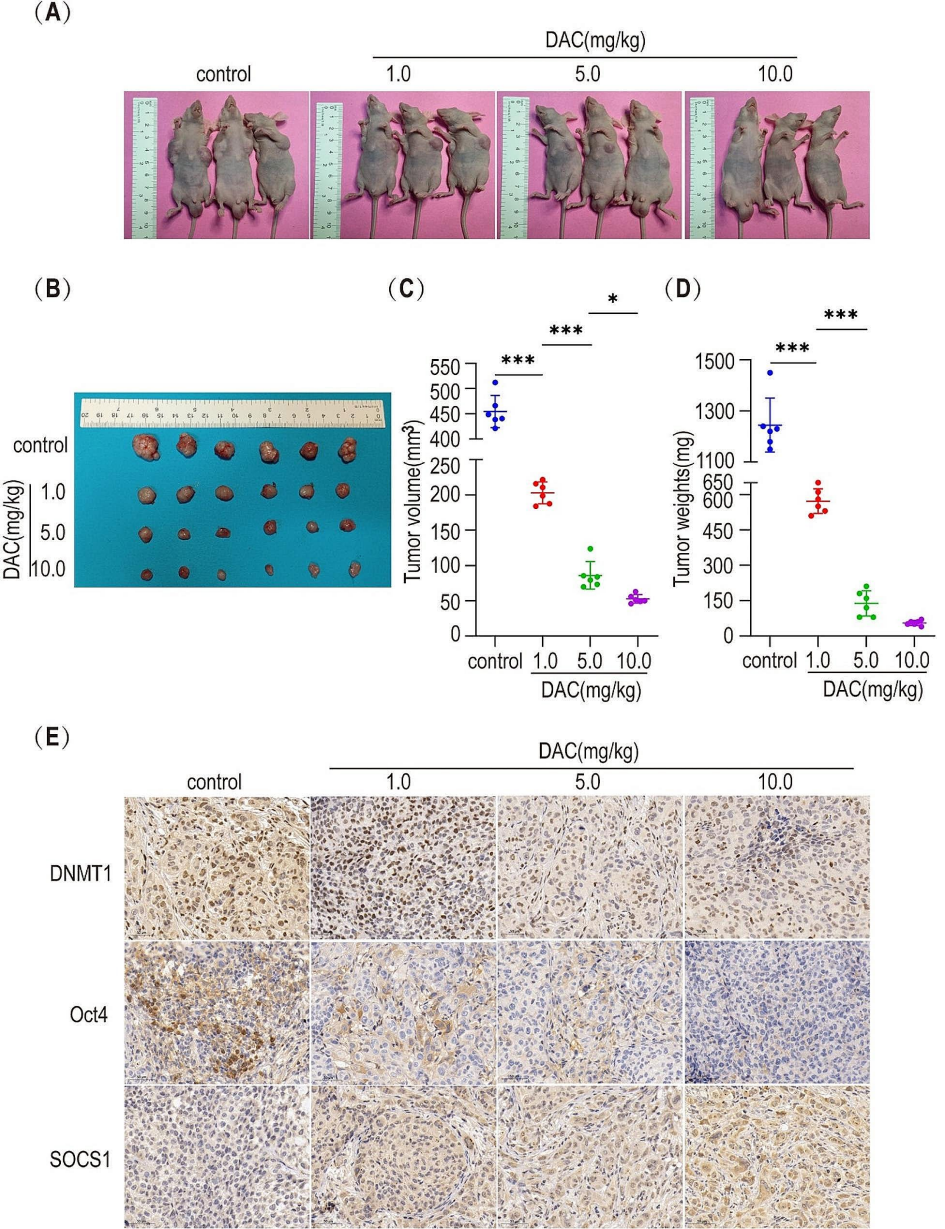
Effect of DNMT1 inhibitors on the growth of xenograft in nude mice. Xenografts from MHCC97H-derived HLCSLCs were treated with different concentrations of DAC. Pre- and post-extraction images of the xenografts (**A**), (**B**) as well as size (**C**) and weight analysis of the tumor (**D**). (**E**) DNMT1, Oct4, and SOCS1 protein expression in tissue sections was analyzed by immunohistochemistry. *p ≤ 0.05, ***p ≤ 0.001

To verify the conclusions obtained from the in vitro experiments, we used the nude mouse xenograft technique for the in vivo experiments. DAC was injected intraperitoneally at varied concentrations to treat MHCC97H-derived HLCSLCs tumor-bearing mice, and the tumor volume and mouse weight were collected using vernier calipers and an electronic balance, respectively. The results suggested that DAC inhibits the growth of xenograft with respect to volume and weight in nude mice in a concentration-dependent manner (Fig. 8A–E). The immunohistochemical staining results of the tissue sections of the xenograft showed a significantly decreased expression of DNMT1 and Oct4 and increased expression of SOCS1 proteins post-DAC treatment (Fig. 8F). These findings suggested that attenuation of DNMT1 expression in nude mice effectively inhibits the growth of xenograft, and the mechanism may be related to DNMT1-regulated methylation of SOCS1 promoter, which in turn alters the stemness of HLCSLCs.

## Discussion

Several studies have shown that epigenetics play a critical role in tumorigenesis, wherein DNA methylation is a natural modification of DNA that is vital for regulating gene expression, embryonic development regulation, genomic imprinting, X chromosome inactivation, and apoptosis [29–32]. It is also a key factor in the development of stem cells towards specific cell and tissue differentiation profiles, and aberrant epigenetic alterations may transform normal stem cells into cancerous stem cells with loss of differentiation ability and acquisition of stem cell-like features [33]. Typically, CpG islands in the promoter region of genes are in a non-methylated state, while DNMT1 is highly expressed in various tumor tissues and catalyzes some tumor suppressor genes to effectuate promoter hypermethylation, resulting in silencing of gene transcripts and loss of normal function, and then exhibiting abnormal biological properties of cells [34–36]. In this study, we used the GEO and TCGA databases and found that DNMT1 was highly expressed in HCC tumor tissues compared to the adjacent normal liver tissues and that patients with high DNMT1 expression had low survival rates. Additionally, HLCSLCs derived from HCC cell lines expressed DNMT1 at a higher level and significantly increased activity compared to their parental cells. This finding suggested that high expression of DNMT1 is associated with maintaining the biological properties of HLCSLCs.

SOCS1 is one of the eight members of the SOCS family of transcription factors, and its central region includes a kinase-inhibitory region that inhibits the activation of JAK2 kinase. SOCS1 is a negative regulator of various inflammatory cytokines and a critical regulator of T cell homeostatic proliferation and differentiation [37]. In recent years, several studies have pointed out a close correlation between SOCS1 and the development of various tumors, especially HCC [38–40]. In addition, SOCS1 methylation was higher than that in paraneoplastic tissues, and the expression was significantly decreased in both HCC tumor samples and HCC cell lines [27]. The methylation status of the *SOCS1* gene in conjunction with serum alpha-fetoprotein (AFP) can be used as a non-invasive biomarker for the diagnosis and prognosis of HCC [39]. These studies suggested that the altered methylation level of the *SOCS1* gene affects its expression during the development and progression of HCC. Herein, we first analyzed the clinical significance of SOCS1 in HCC patients using bioinformatics methods, and the results showed a low expression of SOCS1 in HCC tumor tissues that effectuated a low survival rate of patients. Next, we compared the SOCS1 promoter methylation status and its expression level in parental and parental-derived HLCSLCs and found that HLCSLCs had a higher methylation status and lower expression of SOCS1 promoter. Interestingly, the hypermethylation of SOCS1 promoter in HLCSLCs could be attributed to the direct binding of DNMT1 to the SOCS1 promoter region, which decreases the expression of the molecule; however, this effect can be reversed by DNMT1 inhibitor. Therefore, we proposed that high expression of DNMT1-targeted silencing of SOCS1 expression may be related to the maintenance of stemness of HLCSLCs.

Some studies have shown the presence of HLCSLCs in HCC tissues and cell lines, which are a small group of highly heterogeneous cells with biological properties such as self-renewal, unlimited proliferation, high tumorigenicity, and resistance to radiotherapy and play a crucial role in HCC genesis, progression, metastasis, recurrence, and drug resistance [41–43]. Serum-free suspension culture method combined with stemness characterization is a leading tool for isolating HLCSLCs [44, 45]. In the present study, we evaluated the stemness of HLCSLCs based on four aspects: spheroid formation, soft-agar colony formation, expression of stemness-associated molecules (CD44, Oct4, Nanog, and Sox2), and tumorigenicity of xenograft in nude mice. The results showed that low expression of DNMT1 or overexpression of SOCS1 attenuates the sphere- and soft-agar colony-forming abilities of HLCSLCs; also, the expression of stemness-related molecules was decreased markedly. Moreover, the effect of low expression of SOCS1 in promoting stemness of HLCSLCs could be reversed by DNMT1 inhibitor. The results of the xenograft experiments were consistent with the in vitro experiments.

## Conclusions

Our study clearly demonstrated that the highly expressed and strongly active DNMT1 binds to and catalyzes the SOCS1 promoter region in HLCSLCs, leading to a hypermethylated state, consequently suppressing its expression level, thereby regulating the stemness of HLCSLCs. Taken together, the present study provides a novel idea for the in-depth exploration of the mechanism of stemness maintenance and a new target for the subsequent development of drugs targeting HLCSLCs.

### Electronic supplementary material

Below is the link to the electronic supplementary material.

Supplementary Material 1

Supplementary Material 2

Supplementary Material 3

Supplementary Material 4

Supplementary Material 5

Supplementary Material 6

Supplementary Material 7

Supplementary Material 8

Supplementary Material 9

Supplementary Material 10

Supplementary Material 11

Supplementary Material 12

Supplementary Material 13

Supplementary Material 14

Supplementary Material 15

Supplementary Material 16

Supplementary Material 17

Supplementary Material 18

## Data Availability

All the data obtained and/or analyzed during the current study were available from the corresponding authors on reasonable request.

## Abbreviations

HLCSLCs: Human liver cancer stem-like cells
PCs: parental cells
HCC: hepatocellular carcinoma
ZHX2: Zinc-finger and homeobox 2
KDM2A: lysine demethylase 2 A
H3K36: histone H3 lysine 36
ATO: arsenic trioxide
DNMT1: DNA methyltransferase 1
DAC: decitabine
BEX1: brain-expressed X-linked protein 1
CSCs: stem cells
SOCS1: Suppressor of the cytokine signaling 1
MSP: Methylation-specific PCR
BSP: Bisulfite sequencing PCR
ChIP: Chromatin immunoprecipitation
AFP: alpha-fetoprotein
