# Supplementary figures and images for: Targeted silencing of SOCS1 by DNMT1 promotes stemness of human liver cancer stem-like cells
Source: Cancer Cell Int. 2024 Jun 12;24:206. doi: 10.1186/s12935-024-03322-4 (PMC11170857; doi:10.1186/s12935-024-03322-4)

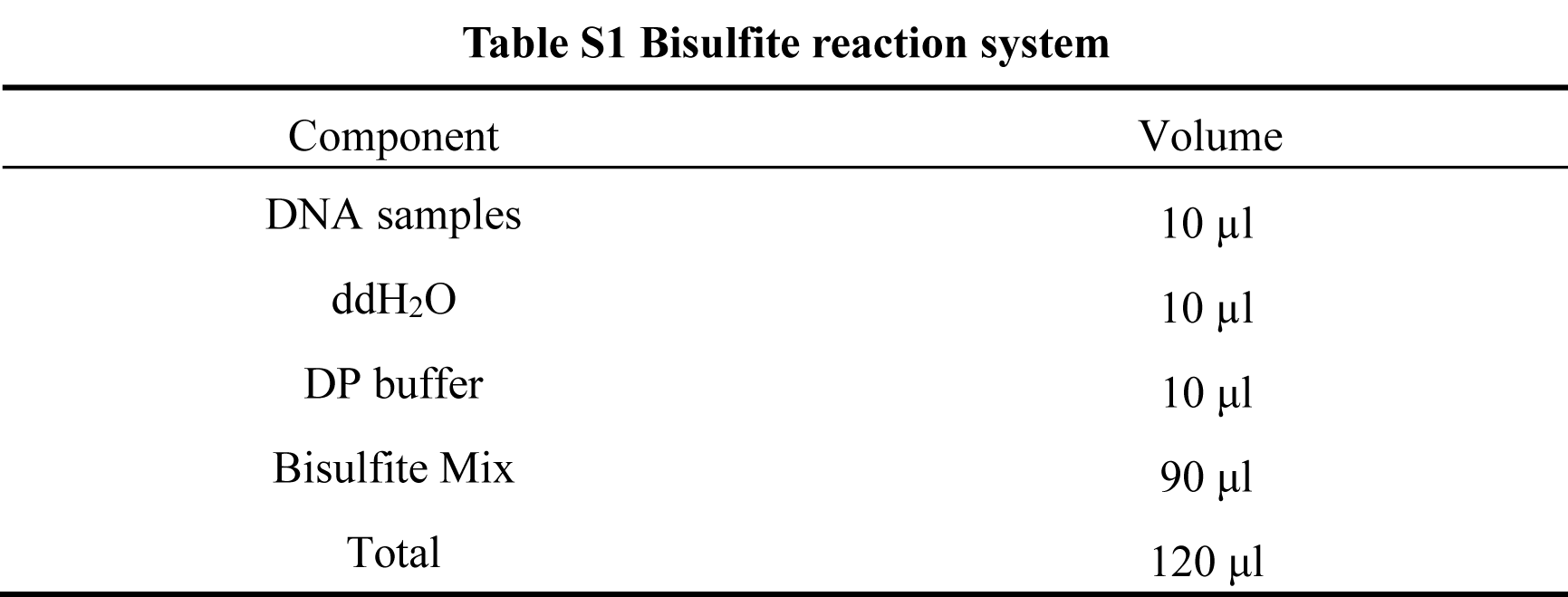

Supplement: Supplementary file 1 — Supplementary Material 1 [file 12935_2024_3322_MOESM1_ESM.tif]

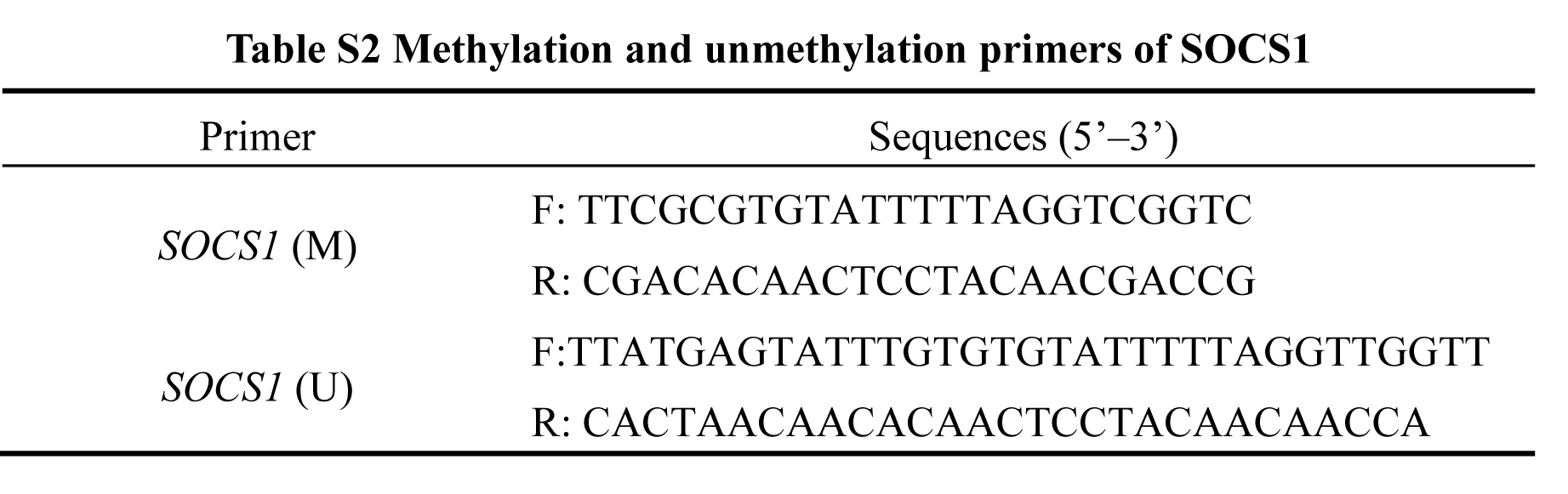

Supplement: Supplementary file 2 — Supplementary Material 2 [file 12935_2024_3322_MOESM2_ESM.tif]

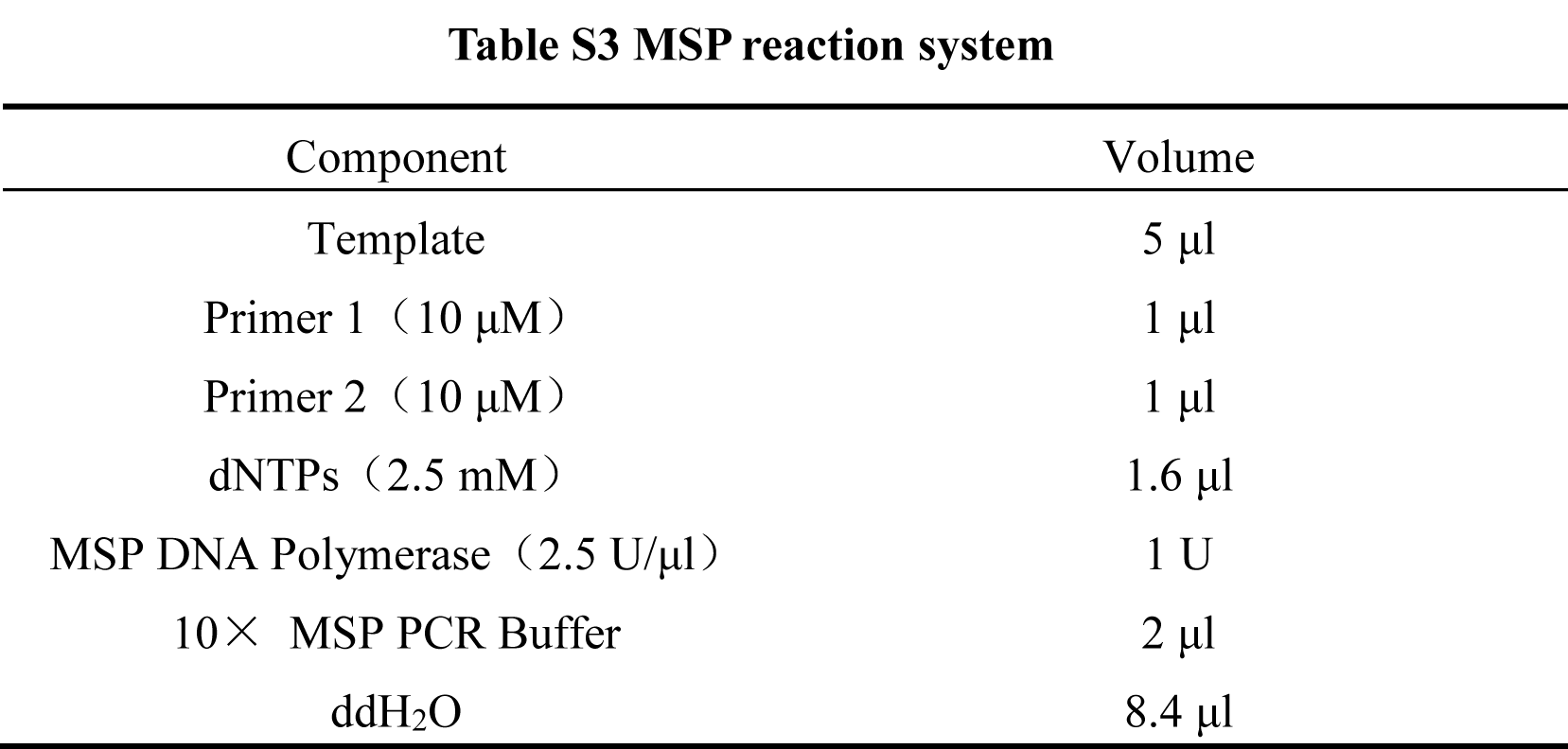

Supplement: Supplementary file 3 — Supplementary Material 3 [file 12935_2024_3322_MOESM3_ESM.tif]

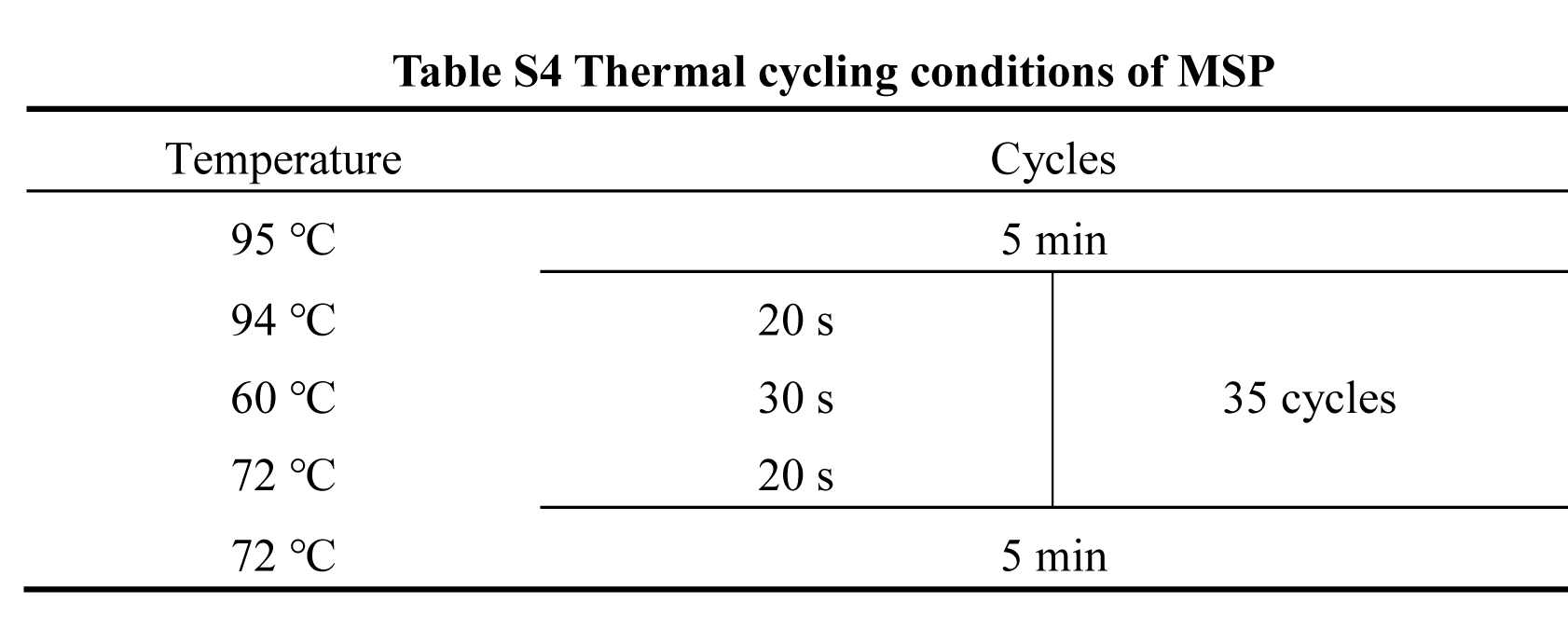

Supplement: Supplementary file 4 — Supplementary Material 4 [file 12935_2024_3322_MOESM4_ESM.tif]

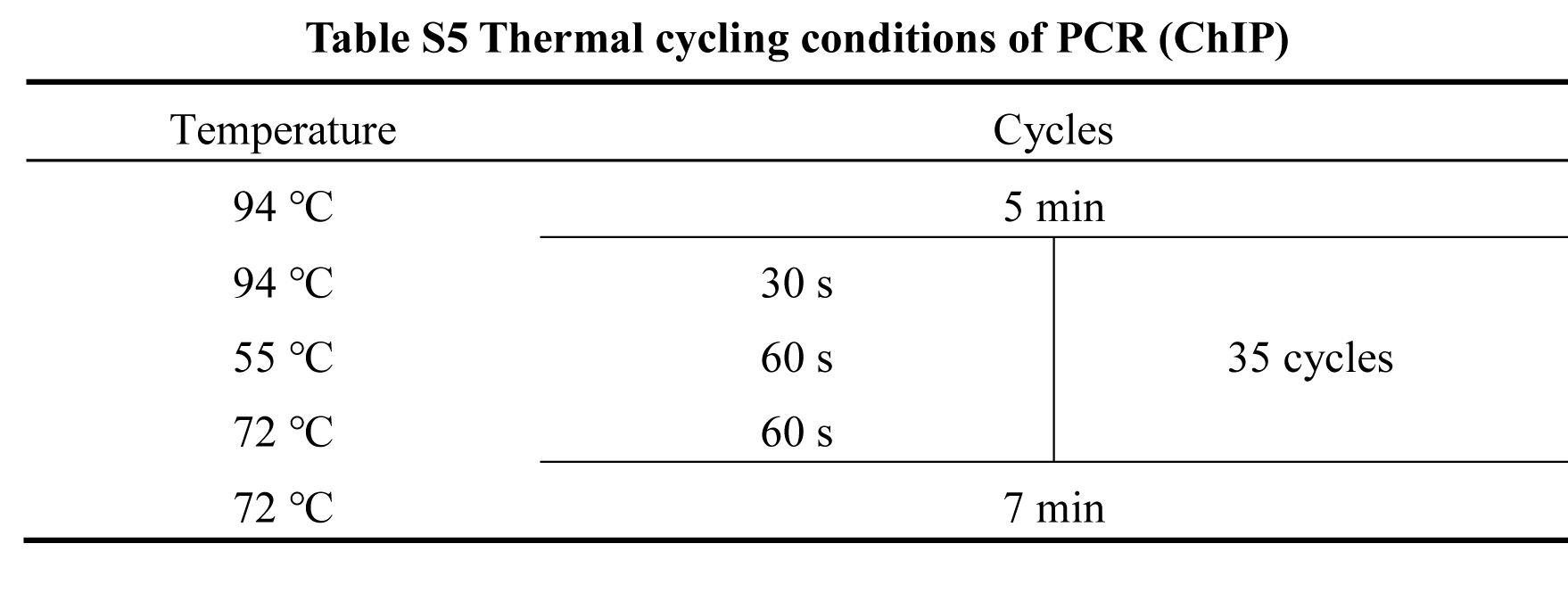

Supplement: Supplementary file 5 — Supplementary Material 5 [file 12935_2024_3322_MOESM5_ESM.tif]

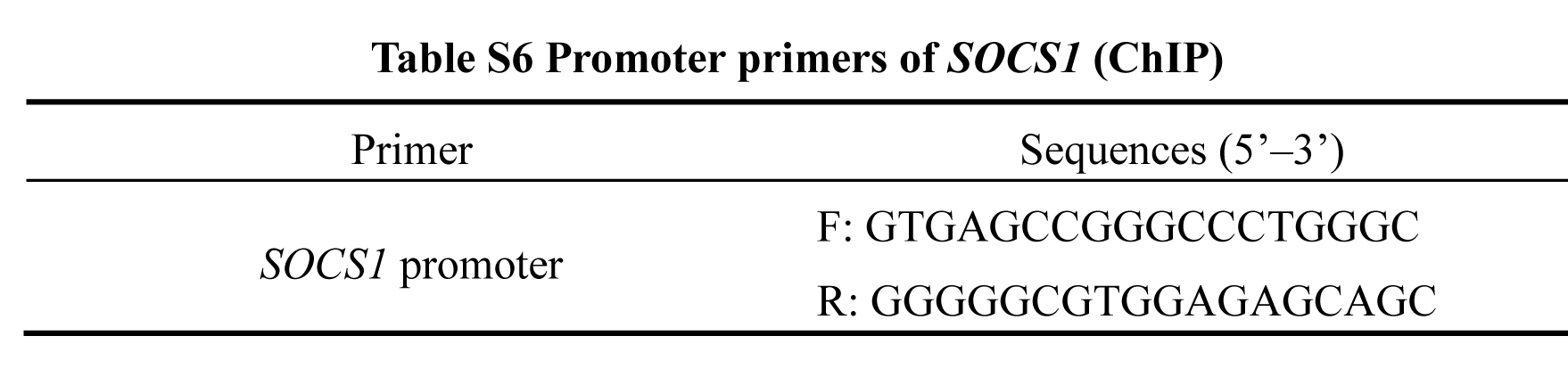

Supplement: Supplementary file 6 — Supplementary Material 6 [file 12935_2024_3322_MOESM6_ESM.tif]

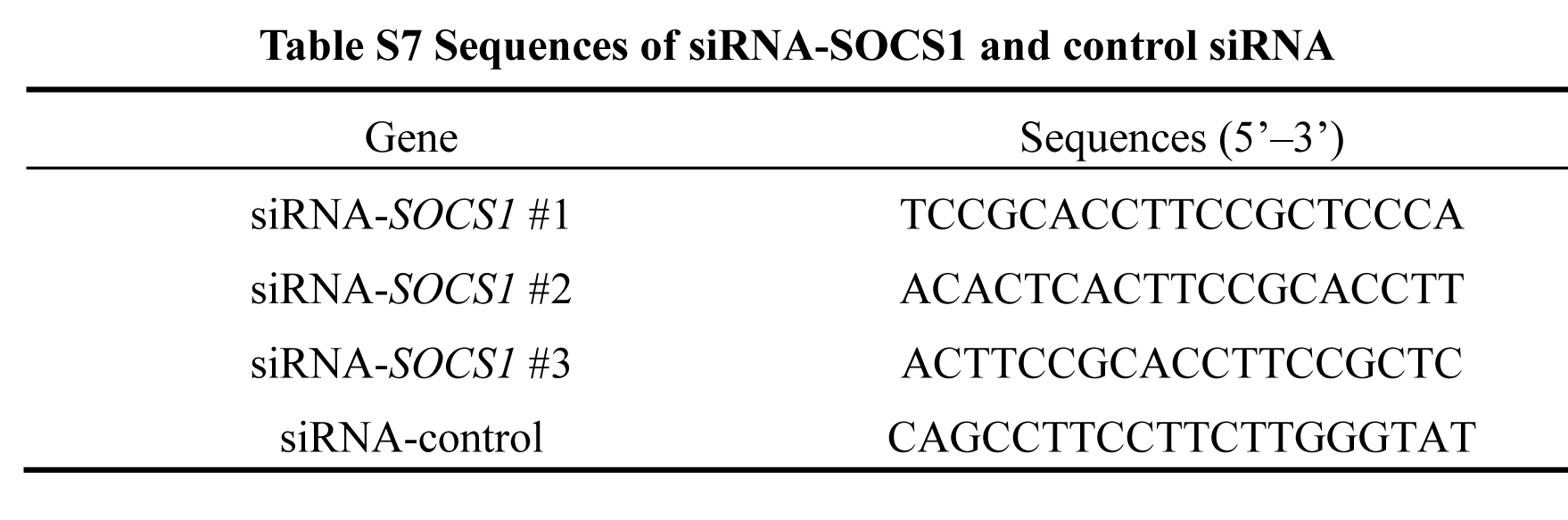

Supplement: Supplementary file 7 — Supplementary Material 7 [file 12935_2024_3322_MOESM7_ESM.tif]

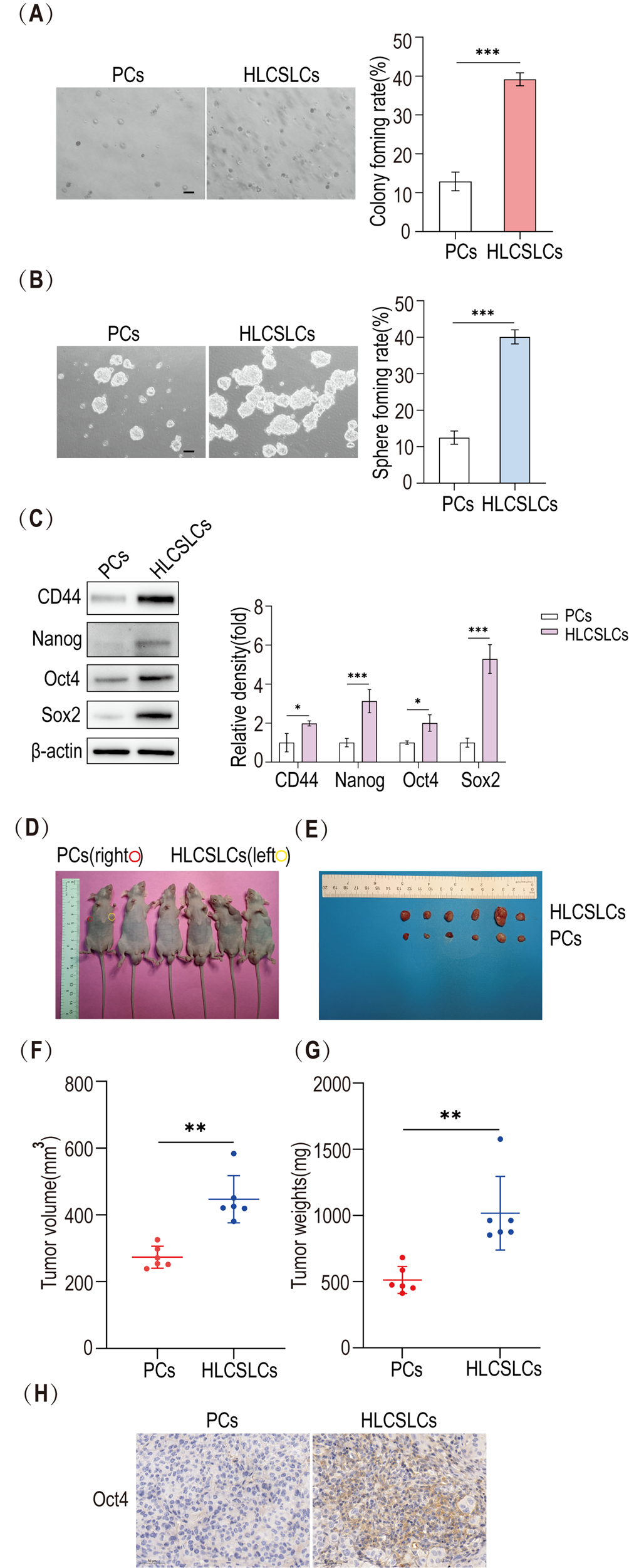

Supplement: Supplementary file 8 — Supplementary Material 8 [file 12935_2024_3322_MOESM8_ESM.tif]

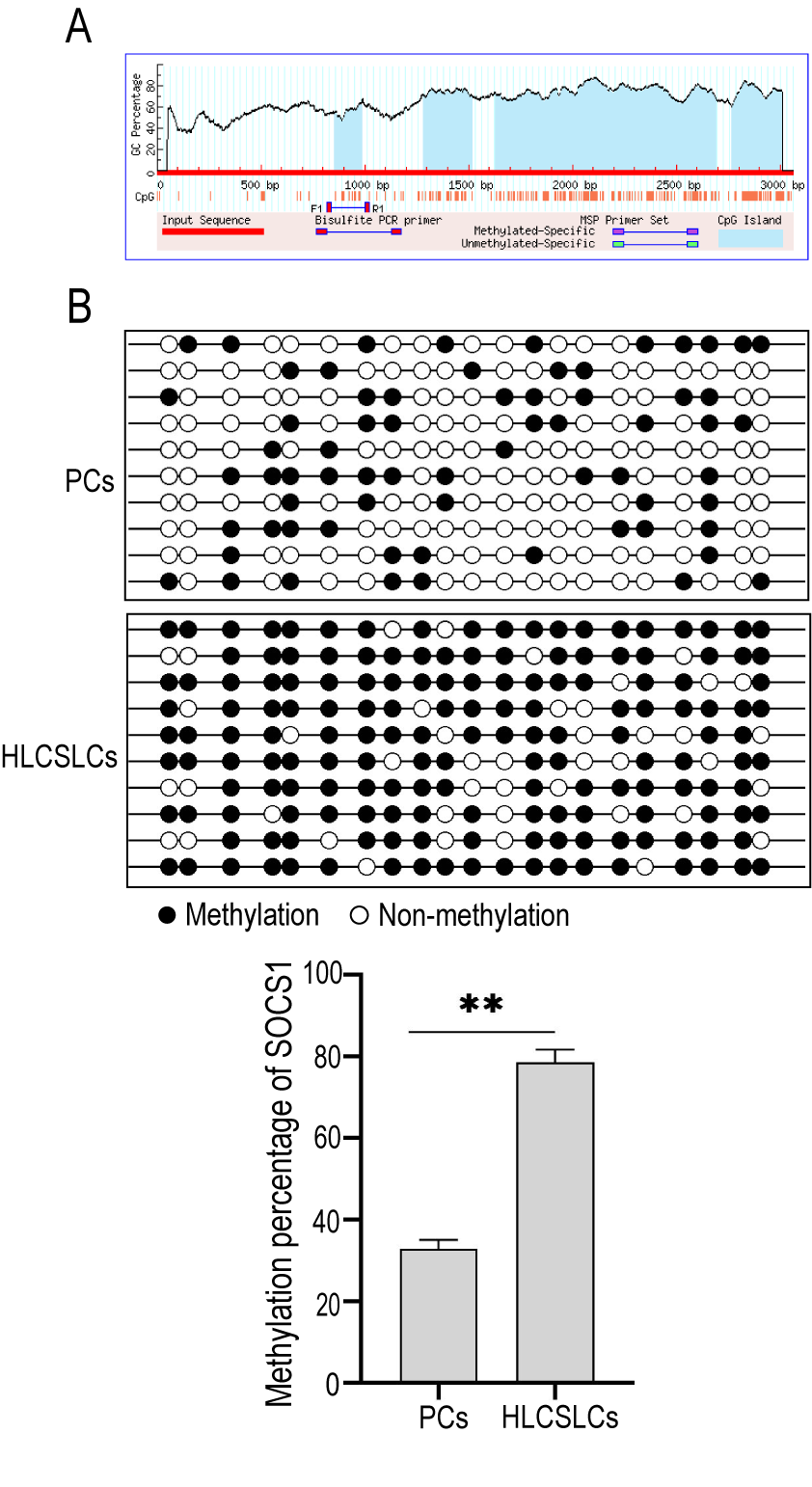

Supplement: Supplementary file 9 — Supplementary Material 9 [file 12935_2024_3322_MOESM9_ESM.tif]

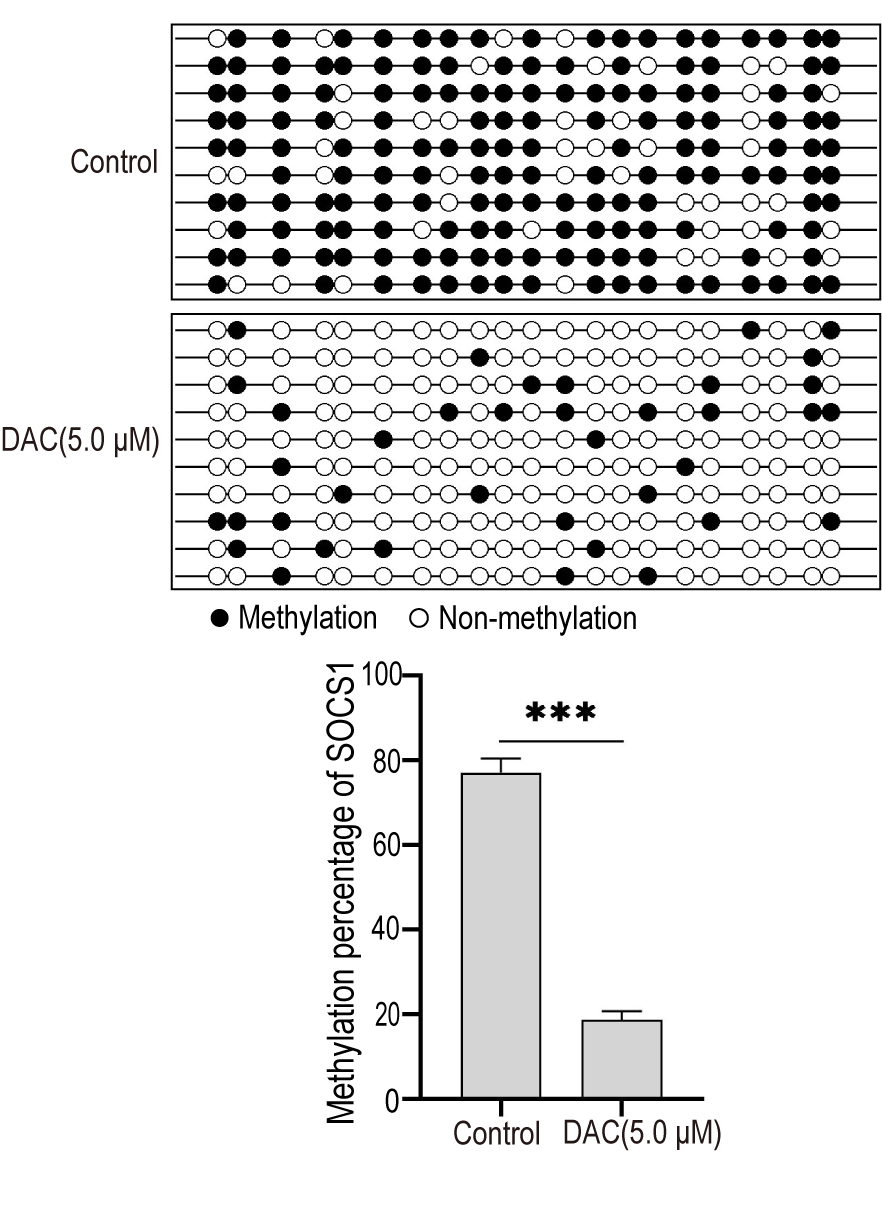

Supplement: Supplementary file 10 — Supplementary Material 10 [file 12935_2024_3322_MOESM10_ESM.tif]

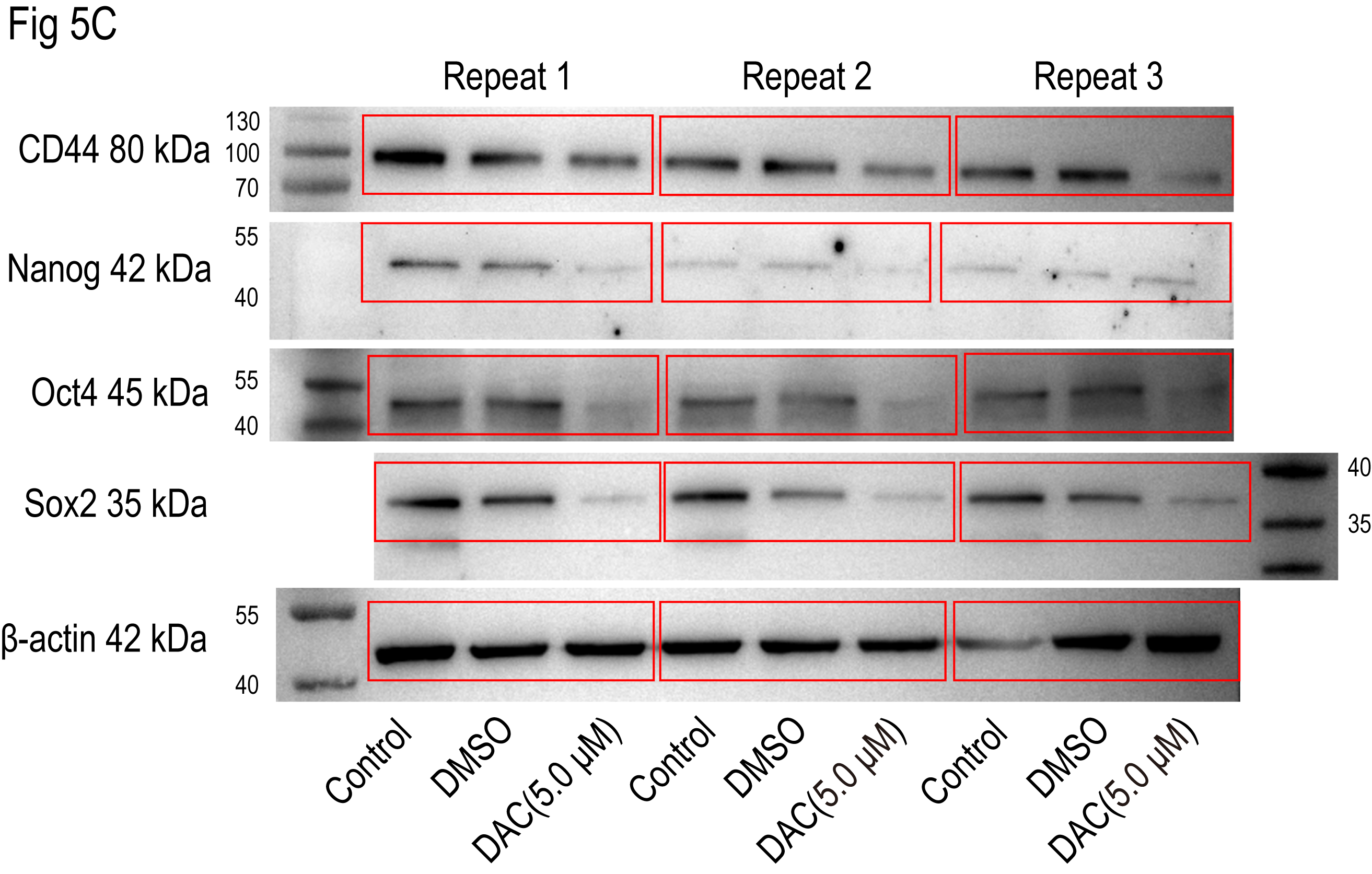

Supplement: Supplementary file 15 — Supplementary Material 15 [file 12935_2024_3322_MOESM15_ESM.tif]

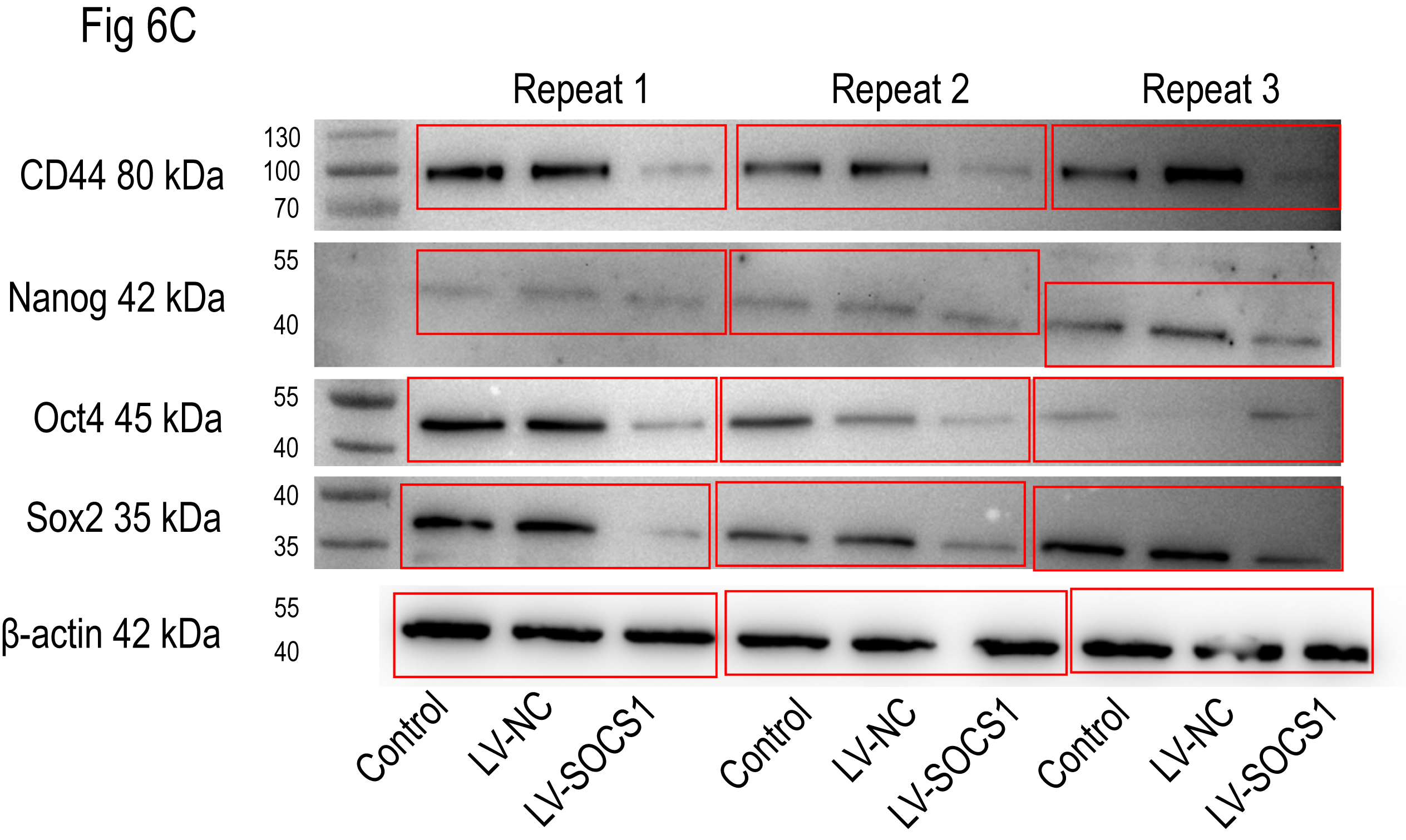

Supplement: Supplementary file 16 — Supplementary Material 16 [file 12935_2024_3322_MOESM16_ESM.tif]

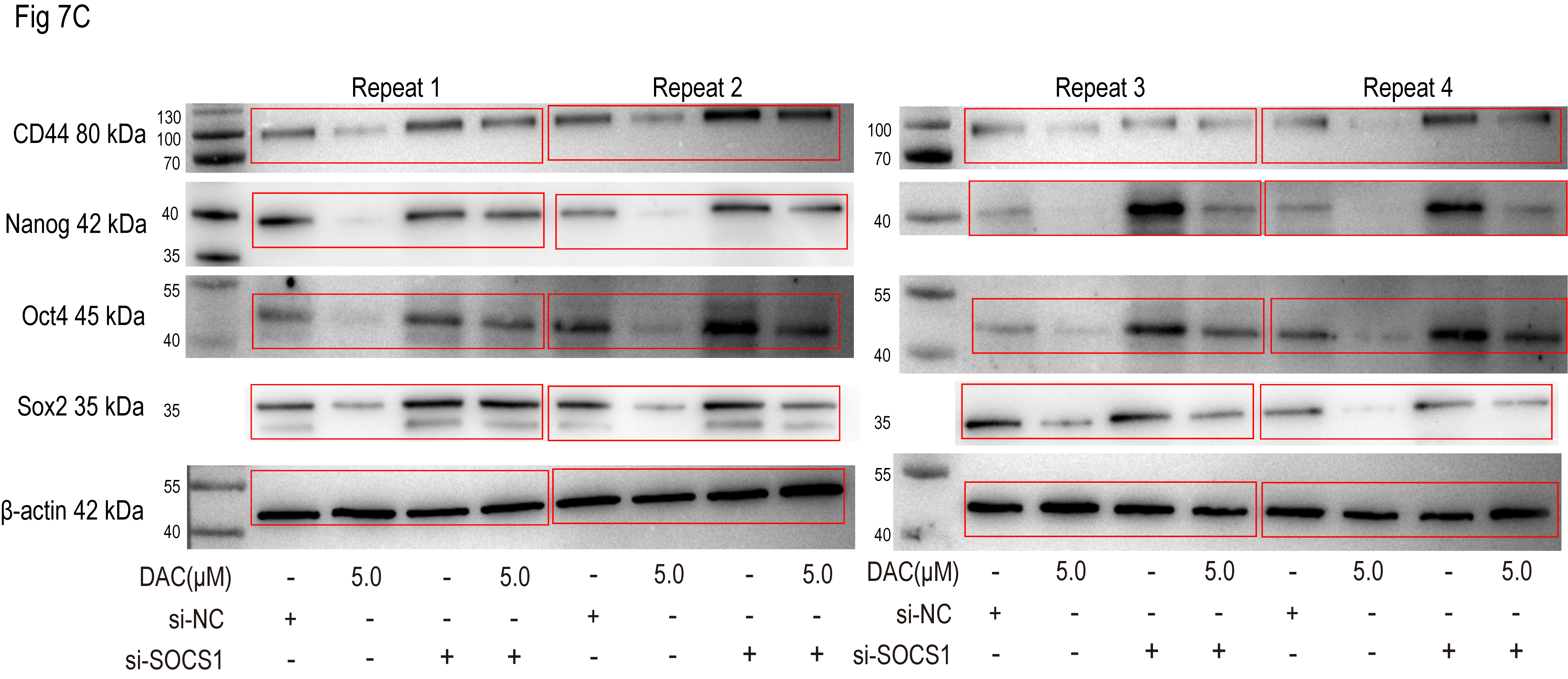

Supplement: Supplementary file 17 — Supplementary Material 17 [file 12935_2024_3322_MOESM17_ESM.tif]

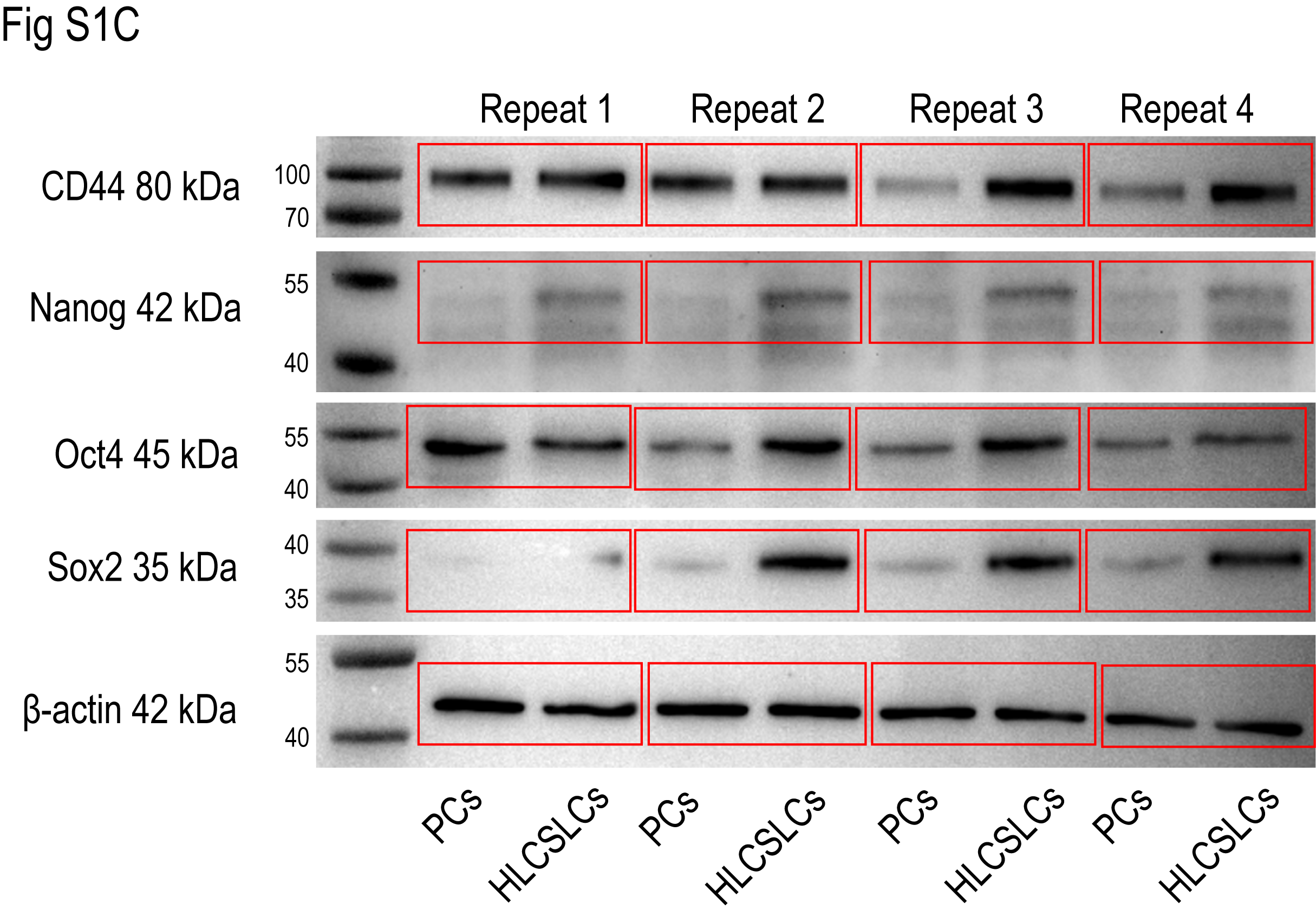

Supplement: Supplementary file 18 — Supplementary Material 18 [file 12935_2024_3322_MOESM18_ESM.tif]
