# Supplementary material for: Targeted silencing of SOCS1 by DNMT1 promotes stemness of human liver cancer stem-like cells
Source: Cancer Cell Int. 2024 Jun 12;24:206. doi: 10.1186/s12935-024-03322-4 (PMC11170857; doi:10.1186/s12935-024-03322-4)
Supplement: Supplementary file 11 — Supplementary Material 11 [file 12935_2024_3322_MOESM11_ESM.docx]

**Fig. S1 Identification of HLCSLCs in follow-up experiments.**

(A) MHCC97H parental cells and MHCC97H-derived HLCSLCs were identified for the ability of colony formation. (B) MHCC97H parental cells and MHCC97H-derived HLCSLCs were identified for the ability of spheroid formation. (C) The expression of stemness-associated factors (CD44, Oct4, Nanog, and Sox2) was determined by Western blot analysis in MHCC97H parental cells and MHCC97H-derived HLCSLCs. (D), (E) Pre- and post-extraction images of the xenografts from MHCC97H parental cells or MHCC97H-derived HLCSLCs. (F), (G) The size and weight analysis of the tumor. (H) Oct4 protein expression in tissue sections was analyzed by immunohistochemistry. **p*≤0.05, ***p*≤0.01, ****p*≤0.001.

**Fig. S2 The methylation status of the SOCS1 promoter in HLCSLCs**

(A) Genomic DNA sequences within the 3-kilobase promoter regions of the SOCS1 gene were analyzed. Four CpG-rich regions (CpG islands) are present the promoter regions of the SOCS1 gene. (B) Bisulfite sequencing PCR (BSP) analysis was performed to compare the methylation status of the SOCS1 promoter in MHCC97H parental cells and MHCC97H-derived HLCSLCs. ***p*≤0.01.

**Fig. S3 The methylation status of the SOCS1 promoter in HLCSLCs treated by DAC (5.0 μM)**

The methylation status of SOCS1 promoter in MHCC97H-derived HLCSLCs treated with DAC (5.0 μM) was determined by Bisulfite sequencing PCR (BSP) analysis. ****p*≤0.001.
