# Supplementary material for: Targeted silencing of SOCS1 by DNMT1 promotes stemness of human liver cancer stem-like cells
Source: Cancer Cell Int. 2024 Jun 12;24:206. doi: 10.1186/s12935-024-03322-4 (PMC11170857; doi:10.1186/s12935-024-03322-4)
Supplement: Supplementary file 12 — Supplementary Material 12 [file 12935_2024_3322_MOESM12_ESM.pdf]

Order: 010-59822688
Toll-free: 800-990-6057 /400-810-6057
TIANGEN BIOTECH (BEIJING) CO., LTD

版本号: DP230630

DNA Bisulfite Conversion Kit

DNA重亚硫酸盐转化试剂盒

（离心柱型）

目录号：DP215

产品内容

产品组成		DP215-02 (50 preps)
DP 215-02	亚硫酸盐干粉（Bisulfite Mix）	10次/管×5支
	缓冲液BM（Buffer BM）	5 ml
	缓冲液DB（Buffer DB）	10 ml
	平衡液BL（Buffer BL）	30 ml
	结合液PB（Buffer PB）	30 ml
	漂洗液PW（Buffer PW）	2×15 ml
	Carrier RNA	310 µg
	RNase-Free ddH$_2$O（管装）	1 ml
	洗脱缓冲液EB（Buffer EB）	15 ml
	吸附柱CB1（Spin Columns CB1）	50 个
	收集管（2 ml）（Collection Tubes 2 ml）	50 个
RK196	缓冲液DP（Buffer DP）	0.5 ml

备注：DP 215-02和RK196组分独立运输和分装。

储存条件

该试剂盒置于室温(15-30℃)干燥条件下，可保存15个月。若溶液产生沉淀，使用前可在37℃水浴中预热10 min以溶解沉淀，不影响效果。缓冲液DP于-30~-15℃下保存，可保存18个月。缓冲液DB加入无水乙醇后于2-8℃下保存，Carrier RNA配制成储液后置于-30~-15℃。

产品简介

表观遗传学是研究在基因的核苷酸序列不发生改变的情况下，基因的表达和调控的可遗传变化的一门遗传学分支学科。表观遗传的现象很多，比如RNA介导的基因沉默，组蛋白修饰等。但是，在高等真核生物中一个主要的表观遗传学机制为DNA的甲基化。

本产品是特别针对DNA甲基化研究中的DNA重亚硫酸盐转化所开发的试剂盒。试剂盒可以在2 h内完成对DNA样品的处理，其中未甲基化胞嘧啶转化为尿嘧啶的转化率高达99%以上。同时，本试剂盒采用独特的DNA保护剂组分，使得转化后DNA的质量和回收率都有很大的保障，另外，本试剂盒还采用了柱上去亚硫酸基团的方法，使得整个操作流程更为简便。

产品特点

简单快速：转化和纯化流程可在2 h内完成，对仪器设备要求较低，适于各级研究机构对DNA样品进行重亚硫酸盐转化。

转化率高：DNA样品中未甲基化胞嘧啶转化为尿嘧啶的转化率高达99%以上。

反应灵敏：本试剂盒可处理少至500 pg，多至2.5 μg的DNA样品。

效果优异：本试剂盒处理后的DNA样品适合于甲基化特异性PCR，测序法及芯片法等后续甲基化分析方法。

注意事项 请务必在使用本试剂盒之前阅读此注意事项。

1. 第一次使用前应按照试剂瓶标签的说明先在15 ml漂洗液PW中加入60 ml无水乙醇，以及在10 ml缓冲液DB中加入30 ml无水乙醇。
2. 若处理的DNA样品少于100 ng， 那么强烈建议将Carrier RNA加入到结合液PB中，至终浓度为10 μg/ml。
3. 所有的离心步骤都为使用台式离心机在室温下进行。
4. 纯化DNA前请使用平衡液BL处理吸附柱，这样可以激活硅基质膜，提高得率。

操作步骤

重亚硫酸盐转化

1. 将待处理的基因组DNA从冰箱中取出解冻，备用。
2. 根据自己的实验要求，配制Bisulfite Mix。配制时，每管干粉中需加入850 μl缓冲液BM，震荡混匀直至干粉完全溶解，整个过程约耗时5 min。

   注意：溶解后的Bisulfite Mix的体积约为950 μl，可供处理10个样品，未用完的Bisulfite Mix可在-15～-30℃下保存1个月。
3. 在200 μl离心管中配制重亚硫酸盐反应体系，具体配制体系如表1所示：

表1 重亚硫酸盐反应体系

组成成分	使用量
DNA样品	X μl (最佳DNA处理量为500-1000 ng)
ddH_2O	20-X μl (DNA与ddH_2O的最大体积为20 μl)
缓冲液 DP	10 μl
Bisulfite Mix	90 μl
Total	120 μl

4. 反应体系配制好以后，在PCR仪等可设置温度变化程序的仪器上进行重亚硫酸盐转化，整个过程约耗时1 h，具体的程序如表2所示：

表2 重亚硫酸盐处理热循环条件

温度	时间
95ºC	10 min
64ºC	30-60 min
4ºC	保持

注意：若初始处理DNA量小于500 ng，只需64℃处理30 min即可，若初始处理DNA量大于 500 ng，则需64℃处理60 min。另外，处理过程中应保证盖紧EP管的帽子，并进行热盖处理。

重亚硫酸盐处理后的DNA纯化

5. 柱平衡步骤：向吸附柱CB1中(吸附柱放入收集管中)加入500 μl的平衡液BL，12,000 rpm (~13,400×g) 离心1 min，倒掉收集管中的废液，将吸附柱重新放回收集管中 **(请使用当天处理过的柱子)**。
6. 重亚硫酸盐处理程序结束后，经过简短离心将管中反应体系转移至干净的1.5 ml 离心管中。
7. 转移好以后，向其中加入5倍体积 (600 μl) 的结合液PB，充分混匀。
8. 将上一步所得溶液加入吸附柱CB1中 (吸附柱放入收集管中)，室温放置2 min，12,000 rpm (~13,400×g)离心30-60 sec，倒掉收集管中的废液，将吸附柱CB1放入收集管中。

   注意：吸附柱容积为800 μl，若样品体积大于800 μl可分批加入。
9. 向吸附柱CB1中加入600 μl漂洗液PW **(使用前请先检查是否已加入无水乙醇)**，12,000 rpm (~13,400×g)离心30-60 sec，倒掉收集管中的废液，将吸附柱CB1放入收集管中。
10. 向吸附柱CB1中加入600 μl溶液DB，室温(15-25℃)放置15 min，12,000 rpm (~13,400×g)离心30-60 sec，倒掉收集管中的废液，将吸附柱CB1放入收集管中。
11. 向吸附柱CB1中加入600 μl漂洗液PW，12,000 rpm (~13,400×g)离心30-60 sec，倒掉收集管中的废液。
12. 重复操作步骤11。
13. 将吸附柱CB1放入收集管中，12,000 rpm (~13,400×g)离心2 min，尽量除去漂洗液。将吸附柱置于室温放置数分钟，彻底地晾干，以防止残留的漂洗液影响下一步的实验。

    注意：漂洗液中乙醇的残留会影响后续的PCR实验。
14. 取出吸附柱CB1，放入一个干净的离心管中，向吸附膜中间位置悬空滴加20 μl洗脱缓冲液EB，室温放置2 min。12,000 rpm(~13,400×g)离心2 min，收集DNA溶液。

    注意：洗脱液的体积不应少于20 μl，体积过少会影响回收效率。为了提高DNA的回收量，可将离心得到的溶液重新加回离心吸附柱中，再次离心。处理后的DNA如果24 h以内即使用，建议保存在2-8ºC中，如需保存更长时间则建议保存在-30~-15ºC中。另外，在后续的PCR扩增反应中，建议在20 μl的反应体系中加入2 μl处理好的基因组DNA作为模板。

DNA浓度及纯度检测

经过重亚硫酸盐处理后的基因组DNA，由于其中非甲基化的“C”会转化为“U”，所以回收后的DNA会是一种“A”，“T”和“U”占大部分的核酸分子，而且当初的碱基配对形式也不复存在，取而代之的是主要以单链形式和非特异性配对形式存在的特殊核酸形态，这种形态的核酸在OD_{260}处的吸光值与RNA更为相似，所以纯化后基因组DNA的OD_{260}值为1时，则相当于大约40 μg/ml的核酸浓度。

另外，基于处理后核酸状态的特殊性，其OD_{230}测值会出现异常现象，可能导致OD_{260}/OD_{230}比值不稳定，实验表明这种情况不会影响后续的PCR反应。

OD_{260}/OD_{280}比值应为1.7-1.9，如果洗脱时不使用洗脱缓冲液，而使用ddH_2O，比值会偏低，因为pH值和离子存在会影响光吸收值，但并不表示纯度低。

浓缩国际权威精华，
铸就TIANGEN优秀品质！

TIANGEN为您提供国际化标准的生物学产品和服务

- PCR、RT-PCR系列
- 核酸DNA、RNA分离纯化系列
- DNA分子量标准
- 克隆载体、感受态细胞
- 细胞生物学产品
- 蛋白分子量标准
- 蛋白质染色、检测及定量相关产品
