# Supplementary material for: Targeted silencing of SOCS1 by DNMT1 promotes stemness of human liver cancer stem-like cells
Source: Cancer Cell Int. 2024 Jun 12;24:206. doi: 10.1186/s12935-024-03322-4 (PMC11170857; doi:10.1186/s12935-024-03322-4)
Supplement: Supplementary file 13 — Supplementary Material 13 [file 12935_2024_3322_MOESM13_ESM.pdf]

TIANGEN®

版本号: EM230630

Methylation-specific PCR (MSP) Kit

甲基化特异性PCR试剂盒

目 录 号: EM101

储存条件: -30~-15℃ 可保存12个月

浓 度: 2.5 U/μl

产品内容:

产品组成	EM101-01
MSP DNA Polymerase (2.5 U/μl)	400 U
10×MSP PCR Buffer	1 ml
dNTPs (2.5 mM)	1 ml

Order: 010-59822688
Toll-free: 800-990-6057/400-810-6057
TIANGEN BIOTECH (BEIJING) CO., LTD.

本产品仅供科研使用。请勿用于医药、临床治疗、食品及化妆品等用途。

产品简介

表观遗传学是研究基因的核苷酸序列不发生改变的情况下，基因表达和调控的可遗传变化的一门遗传学分支学科。表观遗传的现象很多，比如RNA介导的基因沉默、组蛋白修饰等。在高等真核生物中一个主要的表观遗传学机制是DNA的甲基化。

本产品是特别针对通过PCR方法研究基因组DNA甲基化特点的客户所开发的试剂盒。试剂盒组分简单，包含MSP DNA Polymerase，10×MSP PCR Buffe和dNTPs。其中，MSP DNA Polymerase是采用抗体修饰的耐热聚合酶，10×MSP PCR Buffer是特别为MSP反应所优化的PCR缓冲液。本产品具有快速简便、灵敏度高、特异性强、稳定性好等优点。适于与TIANGEN重亚硫酸盐处理试剂盒（DP215-02）搭配使用。

产品组成

1. 10×MSP PCR Buffer：
   500 mM Tris-HCl (pH8.8)
   200 mM KCl
   15 mM $MgCl_2$
   其它稳定剂和增强剂

质量控制

SDS-PAGE检测纯度大于99%；经检测无外源核酸酶活性；能有效地扩增人基因组中的单拷贝基因；室温存放一周，无明显活性改变。

使用说明

本产品使用方便快捷，能避免PCR操作过程中的污染，使用时只需取适量MSP DNA Polymerase，10×MSP PCR Buffer和dNTPs，同时加入模板和引物，并加入ddH$_2$O补足体积，使MSP PCR Buffer的浓度为1×即可进行反应。

适用范围

本产品适用于甲基化特异性PCR（MSP）方法分析基因组DNA的甲基化特点。

反应举例

注意：以下举例仅供参考，实际反应条件因模板、引物等的结构不同而各异，需根据实际情况，设定最佳反应条件。

1. 使用甲基化特异性PCR试剂盒，以重亚硫酸盐处理后的基因组DNA为模板，扩增400 bp的片段，反应体系为20 μl。

组成成分	体 积
Template	< 500 ng
Primer 1（10 μM）	1 μl
Primer 2（10 μM）	1 μl
dNTPs （2.5 mM）	1.6 μl
MSP DNA Polymerase（2.5 U/μl）	1 U
10×MSP PCR Buffer	2 μl
ddH_2O	补至20 μl

2. PCR反应循环的设置：

95°C 5 min

94°C 20 sec
60°C 30 sec　　35 cycles
72°C 20 sec

72°C 5 min

3. 结果检测：反应结束后取10 μl反应产物，琼脂糖凝胶电泳检测。

注意：反复冻融的DNA模板会影响扩增，尽量不要反复冻融DNA模板进行；如需多次实验，可分装后进行冻存，减少冻融次数。
