# Supplementary material for: Targeted silencing of SOCS1 by DNMT1 promotes stemness of human liver cancer stem-like cells
Source: Cancer Cell Int. 2024 Jun 12;24:206. doi: 10.1186/s12935-024-03322-4 (PMC11170857; doi:10.1186/s12935-024-03322-4)
Supplement: Supplementary file 14 — Supplementary Material 14 [file 12935_2024_3322_MOESM14_ESM.pdf]

Order: 010-59822688
Toll-free: 800-990-6057 /400-810-6057
TIANGEN BIOTECH (BEIJING) CO., LTD

版本号: DP210831

TIANamp Genomic DNA Kit

血液/细胞/组织基因组DNA提取试剂盒

（离心柱型）

目录号：DP304

产品内容

产品组成	DP304-02 (50 preps)	DP304-03 (200 preps)
缓冲液GA（Buffer GA）	15 ml	50 ml
缓冲液GB（Buffer GB）	15 ml	50 ml
缓冲液GD（Buffer GD）	13 ml	52 ml
漂洗液PW（Buffer PW）	15 ml	50 ml
洗脱缓冲液TE（Buffer TE）	15 ml	60 ml
Proteinase K	1 ml	4×1 ml
吸附柱CB3（Spin Columns CB3）	50个	200个
收集管（2 ml）（Collection Tubes 2 ml）	50个	200个

选配试剂

红细胞裂解液(Red Cell Lysis Buffer 目录号：RT122)；RNase A（100 mg/ml）（目录号：RT405-12）

储存条件

该试剂盒所有组分置于室温（15-30℃）干燥条件下，可保存15个月。若溶液产生沉淀，使用前可在37℃水浴中预热10min以溶解沉淀，不影响效果。

本产品仅供科研使用。请勿用于医药、临床治疗、食品及化妆品等用途。

产品简介

本试剂盒采用可以特异性结合DNA的离心吸附柱和独特的缓冲液系统，提取多种细胞中的基因组DNA。离心吸附柱中采用的硅基质材料为本公司新型材料，高效、专一吸附DNA，可有效去除杂质蛋白及细胞中其他有机化合物。提取的基因组DNA片段大，纯度高，质量稳定可靠。

使用本试剂盒回收的DNA可适用于各种常规操作，包括酶切、PCR、文库构建、Southern杂交等实验。

提取得率

材料	提取量	DNA得量
哺乳动物全血	100-400 μl	3-10 μg
禽类、两栖类全血	5-20 μl	5-40 μg
动物细胞培养液	10^6-10^7 cells	5-30 μg
动物组织	30 mg	10-30 μg

产品特点：

简单快速： 1 h内即可获得高纯度的基因组DNA。

广　　泛： 适用于血液、多种动物细胞和动物组织等。

纯 度 高： 获得的DNA可直接用于PCR、酶切、杂交等分子生物学实验。

注意事项　<u>请务必在使用本试剂盒之前阅读此注意事项。</u>

1. 第一次使用前应按照试剂瓶标签的说明先在缓冲液GD和漂洗液PW中加入无水乙醇。
2. 样品应避免反复冻融，否则会导致提取的DNA片段较小且提取量也下降。
3. 若缓冲液GA或GB中有沉淀，可在37℃水浴中重新溶解，摇匀后使用。
4. 所有离心步骤均为使用台式离心机，室温下离心。

TIANGEN

操作步骤

使用前请先在缓冲液GD和漂洗液PW中加入无水乙醇，加入体积请参照瓶上的标签。

1. 处理材料

   a. 如提取材料为血液，可直接使用200 μl新鲜、冷冻或加入各种抗凝剂的血液，不足200 μl可加缓冲液GA补足；

   注意：如需处理更大体积血液，如300 μl-1 ml，应按以下步骤操作：在样品中加入3倍体积红细胞裂解液（例如，300 μl血液加入900 μl红细胞裂解液），颠倒混匀，室温放置5 min，期间再颠倒混匀几次。10000 rpm(~11,500×g)离心1 min（若离心机最高转速不允许，可3000 rpm(~3,400×g)离心5 min），吸去上清，留下白细胞沉淀，加200 μl缓冲液GA，振荡至彻底混匀。

   红细胞裂解液本公司另外有售（目录号：RT122），可根据需要来决定购买。

   b. 如果处理血样为禽类、鸟类、两栖类或更低级生物的抗凝血液，其红细胞为有核细胞，因此处理量5-20 μl，可加缓冲液GA补足200 μl后进行下面的裂解步骤。

   c. 贴壁培养的细胞应先处理为细胞悬液，然后10,000 rpm(~11,200×g)离心1 min，倒尽上清，加200 μl缓冲液GA，振荡至彻底悬浮；

   d. 动物组织（脾组织用量应少10 mg）应先打碎处理为细胞悬液，然后10,000 rpm(~11,200×g)离心1 min，倒尽上清，加200 μl缓冲液GA，振荡至彻底悬浮。

   注意：如果需要去除RNA，可加入4 μl RNaseA（100 mg/ml）溶液（客户自备，目录号：RT405-12），振荡15 sec，室温放置5 min。

2. 加入20 μl Proteinase K溶液，混匀。

   a. 提取血液基因组时，只需加入Proteinase K混匀，即可继续进行下一步。

   b. 提取细胞基因组时，只需加入Proteinase K混匀，即可继续进行下一步。

   c. 提取组织基因组时，加入Proteinase K混匀后，在56°C放置，直至组织溶解，简短离心以去除管盖内壁的水珠，再进行下一步骤。

   注意：不同组织裂解时间不同，通常需1-3 h即可完成（鼠尾需要消化过夜）。不会影响后续操作。每小时颠倒混合样品2-3次，用水浴振荡器也可。

3. 加入200 μl缓冲液GB，充分颠倒混匀，70℃放置10 min，溶液应变清亮，简短离心以去除管盖内壁的水珠。

   注意：加入缓冲液GB时可能会产生白色沉淀，一般70℃放置时会消失，不会影响后续实验。如溶液未变清亮，说明细胞裂解不彻底，可能导致提取DNA量少和提取出的DNA不纯。当血液体积≤200 μl且没有采用红细胞裂解处理，或是样本储存条件不佳，水浴后颜色可能为深褐色，注意溶液中没有团块等沉淀。

4. 加入200 μl 无水乙醇，充分振荡混匀15 sec，此时可能会出现絮状沉淀，简短离心以去除管盖内壁的水珠。

5. 将上一步所得溶液和絮状沉淀都加入一个吸附柱CB3中（**吸附柱放入收集管中**），12,000 rpm (~13,400×g)离心30 sec，倒掉废液，将吸附柱CB3放回收集管中。

6. 向吸附柱CB3中加入500 μl缓冲液GD（**使用前请先检查是否已加入无水乙醇**），12,000 rpm (~13,400×g)离心30 sec，倒掉废液，将吸附柱CB3放入收集管中。

7. 向吸附柱CB3中加入600 μl 漂洗液PW（**使用前请先检查是否已加入无水乙醇**），12,000 rpm (~13,400×g)离心30 sec，倒掉废液，将吸附柱CB3放入收集管中。

8. 重复操作步骤7。

9. 将吸附柱CB3放回收集管中，12,000 rpm(~13,400×g)离心2 min，倒掉废液。将吸附柱CB3置于室温放置数分钟，以彻底晾干吸附材料中残余的漂洗液。

   注意：这一步的目的是将吸附柱中残余的漂洗液去除，漂洗液中乙醇的残留会影响后续的酶反应（酶切、PCR等）实验。

10. 将吸附柱CB3转入一个干净的离心管中，向吸附膜的中间部位悬空滴加50-200 μl洗脱缓冲液TE，室温放置2-5 min，12,000 rpm (~13,400×g)离心 2 min，将溶液收集到离心管中。

    注意：洗脱缓冲液体积不应少于50 μl，体积过小影响回收效率。洗脱液的pH值对于洗脱效率有很大影响。若用ddH$_2$O做洗脱液应保证其pH值在7.0-8.5范围内，pH值低于7.0会降低洗脱效率；且DNA产物应保存在-20℃，以防DNA降解。为增加基因组DNA的得率，可将离心得到的溶液再加入吸附柱CB3中，室温放置2 min，12,000 rpm (~13,400×g)离心2 min。
